# Supplementary material for: Effects of Clinical Wastewater on the Bacterial Community Structure from Sewage to the Environment
Source: Microorganisms. 2021 Mar 31;9(4):718. doi: 10.3390/microorganisms9040718 (PMC8065902; doi:10.3390/microorganisms9040718)
Supplement: Supplementary file 1 [file microorganisms-09-00718-s001.zip › reviewer link for SRA.docx]

reviewer link for SRA:

BioProject PRJNA668064:

<https://dataview.ncbi.nlm.nih.gov/object/PRJNA668064?reviewer=lnjpkerfcarqgeemj1seq2f0qi>

BioProject PRNJA668059

<https://dataview.ncbi.nlm.nih.gov/object/PRJNA668059?reviewer=ahfgak3p2b2cq2hlv8pkk59i4u>
