# Supplementary material for: Effects of Clinical Wastewater on the Bacterial Community Structure from Sewage to the Environment
Source: Microorganisms. 2021 Mar 31;9(4):718. doi: 10.3390/microorganisms9040718 (PMC8065902; doi:10.3390/microorganisms9040718)
Supplement: Supplementary file 1 [file microorganisms-09-00718-s001.zip › Supplementary material.docx]

Supplementary material


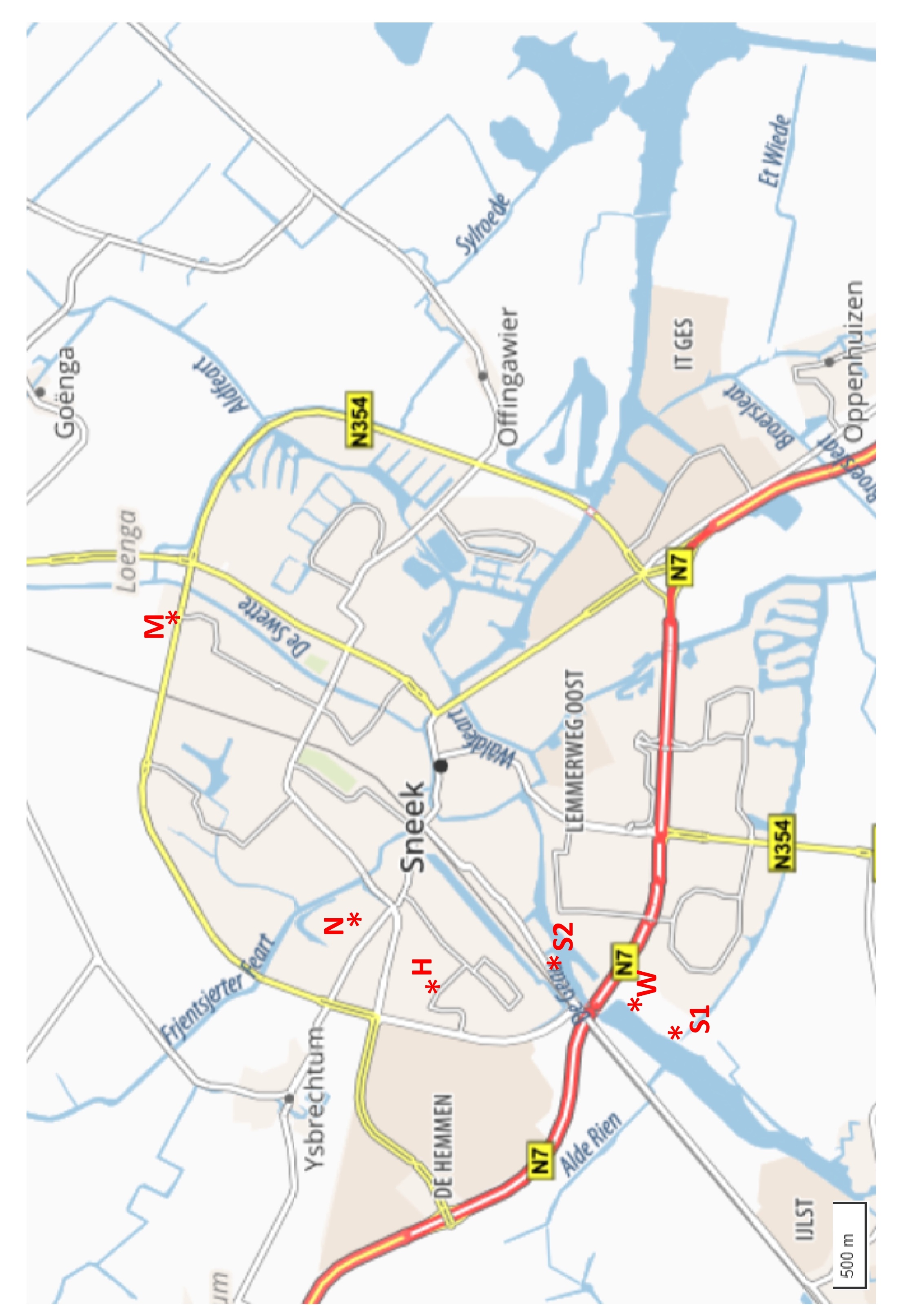


**Figure S1. Locations of the sampling points in Sneek.** H = hospital, N = nursing home, C = community, W = WWTP (influent and effluent), S1 and S2 = Receiving surface water. The location of the control surface water sample is not indicated. The map of Sneek is obtained from viamichelin.nl.

**Table S1a. Statistical analysis of weighted UniFrac distances along the wastewater pathway**. The p-values of the PERMANOVA test are shown below the diagonal (Adjustment method for p-value: FDR), and the permuted p-values using permutation test for homogeneity of multivariate dispersion are shown above the diagonal.

|  | **Hospital** | **Nursing-home** | **Community** | **Influent** | **Effluent** |
| --- | --- | --- | --- | --- | --- |
| **Hospital** | X | 0.800 | 0.233 | 0.218 | 0.775 |
| **Nursing-home** | **0.001** | X | 0.201 | 0.439 | 0.605 |
| **Community** | **0.001** | **0.001** | X | **0.010** | 0.461 |
| **Influent** | **0.001** | **0.001** | **0.001** | X | 0.115 |
| **Effluent** | **0.001** | **0.001** | **0.001** | **0.001** | X |

**Table S1b. Statistical analysis of unweighted UniFrac distances along the wastewater pathway. The** p-values of the PERMANOVA test are shown below the diagonal (Adjustment method for p-value: FDR), and the permuted p-values using permutation test for homogeneity of multivariate dispersion are shown above the diagonal.

|  | **Hospital** | **Nursing-home** | **Community** | **Influent** | **Effluent** |
| --- | --- | --- | --- | --- | --- |
| **Hospital** | X | **0.014** | **0,009** | **0.018** | **0.001** |
| **Nursing-home** | **0.001** | X | 0.554 | 0.684 | **0.001** |
| **Community** | **0.001** | **0.001** | X | 0.970 | **0.001** |
| **Influent** | **0.001** | **0.001** | **0.001** | X | **0.001** |
| **Effluent** | **0.001** | **0.001** | **0.001** | **0.001** | X |

| 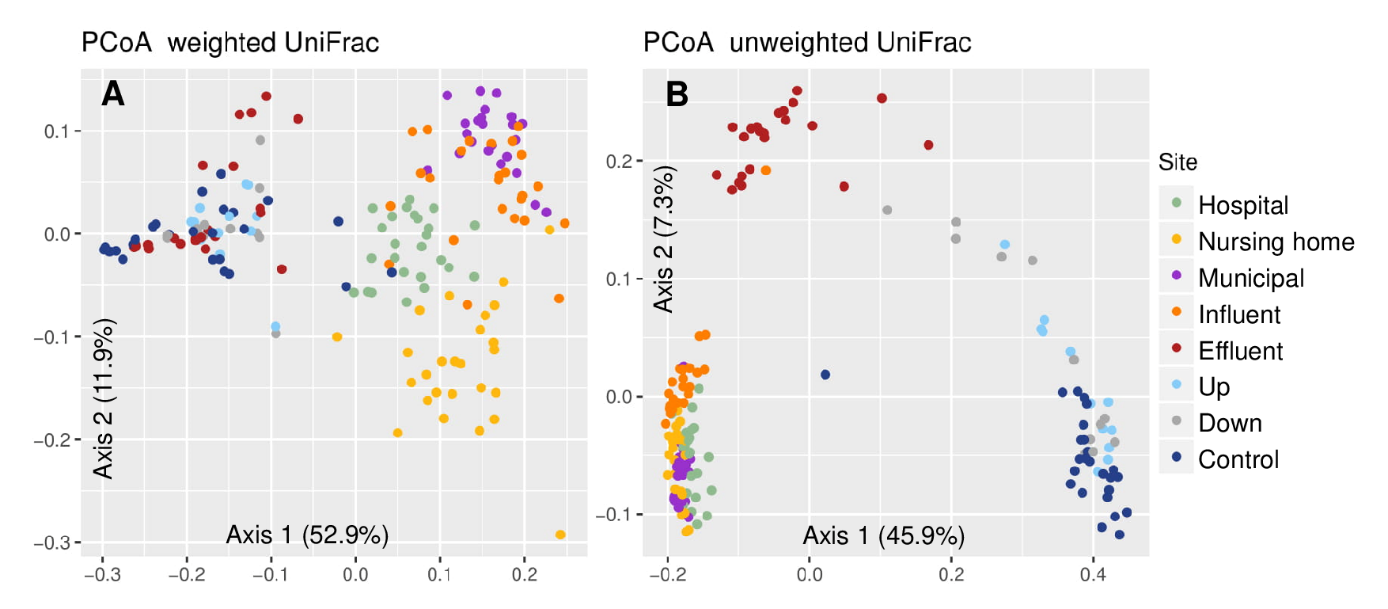 |
| --- |

**Figure S2. Bacterial beta diversity off all the locations.** Principal coordinates ordination of different wastewaters based on weighted UniFrac distances (A) and unweighted UniFrac distances (B). 69% of the diversity in bacterial composition and 59% of the diversity in bacterial membership could be explained by the locations. The percentage of variation explained by each axis is shown between parentheses. H = hospital, N = nursing home, C = community, I = influent, E = effluent, Up = upstream surface water, Down = downstream surface water, and Control = control surface water.

| **Table S2a. Statistical analysis of weighted UniFrac distances along all the locations.** The p-values of the PERMANOVA test are shown below the diagonal (Adjustment method for p-value: FDR), and the permuted p-values using permutation test for homogeneity of multivariate dispersion are shown above the diagonal.   \|  \| **Hospital** \| **Nursing-home** \| **Community** \| **Influent** \| **Effluent** \| **Up-stream** \| \| **Down-stream** \| **Control** \| \| --- \| --- \| --- \| --- \| --- \| --- \| --- \| --- \| --- \| --- \| \| **Hospital** \| X \| 0.784 \| 0.219 \| 0.202 \| 0.565 \| 0.742 \| 0.886 \| \| **0.024** \| \| **Nursing-home** \| **0.001** \| X \| 0.215 \| 0.419 \| 0.448 \| 0.605 \| 0.927 \| \| 0.090 \| \| **Community** \| **0.001** \| **0.001** \| X \| **0.007** \| 0.771 \| 0.525 \| 0.275 \| \| **0.004** \| \| **Influent** \| **0.001** \| **0.001** \| **0.001** \| X \| 0.121 \| 0.141 \| 0.397 \| \| 0.162 \| \| **Effluent** \| **0.001** \| **0.001** \| **0.001** \| **0.001** \| X \| 0.896 \| 0.613 \| \| **0.023** \| \| **Up-stream** \| **0.001** \| **0.001** \| **0.001** \| **0.001** \| **0.001** \| X \| 0.717 \| \| 0.077 \| \| **Down-stream** \| **0.001** \| **0.001** \| **0.001** \| **0.001** \| **0.001** \| 0.967 \| X \| \| 0.132 \| \| **Control** \| **0.001** \| **0.001** \| **0.001** \| **0.001** \| **0.001** \| **0.042** \| 0.085 \| \| X \| |
| --- | --- | --- | --- | --- | --- | --- | --- | --- | --- | --- | --- | --- | --- | --- | --- | --- | --- | --- | --- | --- | --- | --- | --- | --- | --- | --- | --- | --- | --- | --- | --- | --- | --- | --- | --- | --- | --- | --- | --- | --- | --- | --- | --- | --- | --- | --- | --- | --- | --- | --- | --- | --- | --- | --- | --- | --- | --- | --- | --- | --- | --- | --- | --- | --- | --- | --- | --- | --- | --- | --- | --- | --- | --- | --- | --- | --- | --- | --- | --- | --- | --- | --- | --- | --- | --- | --- | --- | --- | --- | --- |
| **Table S2b. Statistical analysis of unweighted UniFrac distances along all the locations.** The p-values of the PERMANOVA test are shown below the diagonal (Adjustment method for p-value: FDR), and the permuted p-values using permutation test for homogeneity of multivariate dispersion are shown above the diagonal.   \|  \| **Hospital** \| **Nursing-home** \| **Community** \| **Influent** \| **Effluent** \| **Up-stream** \| \| **Down-stream** \| **Control** \| \| --- \| --- \| --- \| --- \| --- \| --- \| --- \| --- \| --- \| --- \| \| **Hospital** \| X \| **0.024** \| **0.002** \| **0.019** \| **0.001** \| **0.001** \| **0.001** \| \| **0.001** \| \| **Nursing-home** \| **0.001** \| X \| 0.542 \| 0.678 \| **0.001** \| **0.001** \| **0.001** \| \| **0.001** \| \| **Community** \| **0.001** \| **0.001** \| X \| 0.962 \| **0.001** \| **0.001** \| **0.001** \| \| **0.001** \| \| **Influent** \| **0.001** \| **0.001** \| **0.001** \| X \| **0.001** \| **0.001** \| **0.001** \| \| **0.001** \| \| **Effluent** \| **0.001** \| **0.001** \| **0.001** \| **0.001** \| X \| **0.002** \| **0.001** \| \| **0.001** \| \| **Up-stream** \| **0.001** \| **0.001** \| **0.001** \| **0.001** \| **0.001** \| X \| 0.329 \| \| 0.819 \| \| **Down-stream** \| **0.001** \| **0.001** \| **0.001** \| **0.001** \| **0.001** \| 0.754 \| X \| \| 0.467 \| \| **Control** \| **0.001** \| **0.001** \| **0.001** \| **0.001** \| **0.001** \| **0.001** \| **0.001** \| \| X \| |

|  |
| --- |
| 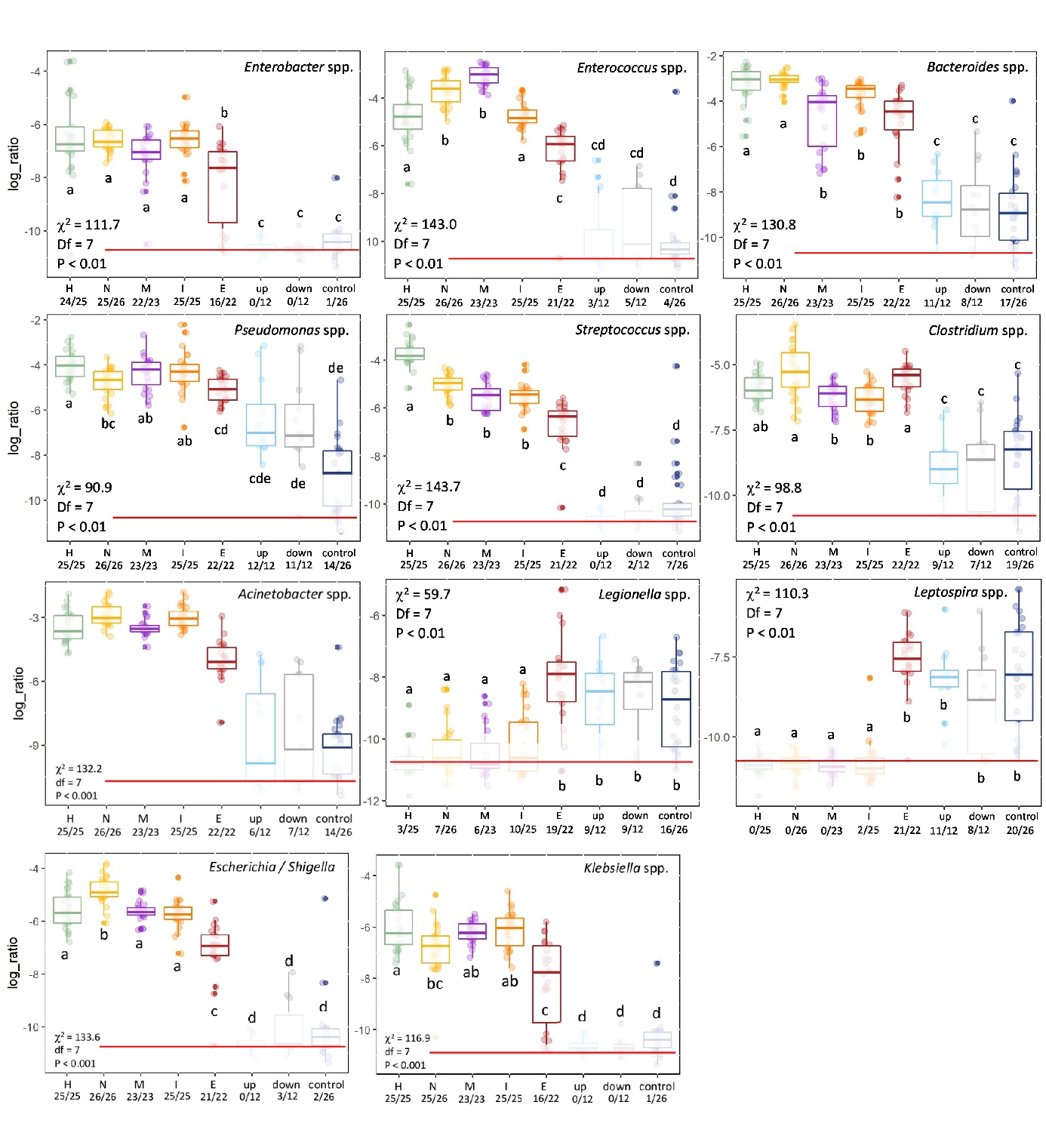 |
| **Figure S3. Target genera based on pathogenic potential.** Relative abundance of target genera along the wastewater pathway. The Kruskal-Wallis statistics are shown on the left side of each panel, for all species there was significant difference in abundance observed between two or more locations. Group differences were assessed Dunn’s test with P value adjustment method: BH. The red line represents the average detection limit. Samples below the detection limit are faded. H = hospital, N = nursing home, M = municipal, I = influent, E = effluent, up = upstream surface water, down = downstream surface water, and control = control surface water. |

**Table S3. Clinical enriched bacteria.** 207 taxa were identified to be significantly more abundant in both hospital (H) and nursing-home (N) wastewater when compared to community (C) wastewater. Taxa belonging to the target genera that include potential pathogenic genera are shown in red.

| **Bacterial identification** | | | | | **Mean abundance (%)** | | | **Log2-FoldChange** | | **p-value** | |
| --- | --- | --- | --- | --- | --- | --- | --- | --- | --- | --- | --- |
| **Class** | **Order** | **Family** | **Genus** | **Species** | **H** | **N** | **C** | **H - C** | **N - C** | **H - C** | **N - C** |
| Actinobacteria | Actinomycetales | Actinomycetaceae | Actinomyces | NA | 0,041 | 0,069 | 0,001 | 3,402 | 3,983 | 0,000 | 0,000 |
| Actinobacteria | Micrococcales | Bogoriellaceae | Georgenia | NA | 0,012 | 0,016 | 0,001 | 3,071 | 3,595 | 0,000 | 0,000 |
| Actinobacteria | Micrococcales | Micrococcaceae | Glutamicibacter | NA | 0,018 | 0,013 | 0,003 | 2,637 | 2,239 | 0,020 | 0,058 |
| Actinobacteria | NA | NA | NA | NA | 0,005 | 0,028 | 0,000 | 2,190 | 4,115 | 0,020 | 0,000 |
| Actinobacteria | Propionibacteriales | Propionibacteriaceae | Propioniciclava | NA | 0,023 | 0,010 | 0,000 | 3,581 | 2,551 | 0,005 | 0,063 |
| Coriobacteriia | Coriobacteriales | Coriobacteriaceae | Enterorhabdus | NA | 0,027 | 0,027 | 0,028 | 0,635 | 0,702 | 0,075 | 0,043 |
| Coriobacteriia | Coriobacteriales | Coriobacteriaceae | Gordonibacter | NA | 0,020 | 0,032 | 0,003 | 2,737 | 3,133 | 0,000 | 0,000 |
| Coriobacteriia | Coriobacteriales | Coriobacteriaceae | NA | NA | 0,004 | 0,009 | 0,001 | 2,066 | 2,858 | 0,037 | 0,001 |
| Bacteroidia | Bacteroidales | Bacteroidaceae | Bacteroides | clarus | 0,017 | 0,044 | 0,000 | 3,086 | 4,277 | 0,000 | 0,000 |
| Bacteroidia | Bacteroidales | Bacteroidaceae | Bacteroides | coprocola | 0,067 | 0,072 | 0,013 | 1,890 | 1,761 | 0,000 | 0,001 |
| Bacteroidia | Bacteroidales | Bacteroidaceae | Bacteroides | eggerthii | 0,062 | 0,064 | 0,007 | 2,193 | 2,443 | 0,000 | 0,000 |
| Bacteroidia | Bacteroidales | Bacteroidaceae | Bacteroides | NA | 0,046 | 0,092 | 0,001 | 3,964 | 4,522 | 0,000 | 0,000 |
| Bacteroidia | Bacteroidales | Bacteroidaceae | Bacteroides | NA | 0,232 | 0,187 | 0,015 | 3,292 | 3,031 | 0,000 | 0,000 |
| Bacteroidia | Bacteroidales | Bacteroidaceae | Bacteroides | NA | 0,044 | 0,273 | 0,003 | 3,050 | 3,883 | 0,000 | 0,000 |
| Bacteroidia | Bacteroidales | Bacteroidaceae | Bacteroides | NA | 0,068 | 0,060 | 0,012 | 2,140 | 2,063 | 0,001 | 0,002 |
| Bacteroidia | Bacteroidales | Bacteroidaceae | Bacteroides | NA | 0,074 | 0,072 | 0,017 | 1,639 | 1,819 | 0,000 | 0,000 |
| Bacteroidia | Bacteroidales | Bacteroidaceae | Bacteroides | NA | 0,117 | 0,122 | 0,031 | 1,225 | 1,313 | 0,000 | 0,000 |
| Bacteroidia | Bacteroidales | Bacteroidaceae | Bacteroides | NA | 0,350 | 0,371 | 0,096 | 0,943 | 1,080 | 0,000 | 0,000 |
| Bacteroidia | Bacteroidales | Bacteroidaceae | Bacteroides | stercoris | 0,114 | 0,067 | 0,002 | 4,068 | 4,095 | 0,000 | 0,000 |
| Bacteroidia | Bacteroidales | Bacteroidaceae | Bacteroides | thetaiotaomicron | 0,091 | 0,077 | 0,008 | 2,399 | 2,469 | 0,000 | 0,000 |
| Bacteroidia | Bacteroidales | Bacteroidaceae | Bacteroides | uniformis | 0,174 | 0,316 | 0,063 | 1,158 | 1,382 | 0,000 | 0,000 |
| Bacteroidia | Bacteroidales | Bacteroidaceae | Bacteroides | uniformis | 0,271 | 0,408 | 0,053 | 1,143 | 1,384 | 0,000 | 0,000 |
| Bacteroidia | Bacteroidales | Bacteroidaceae | Bacteroides | vulgatus | 0,245 | 0,377 | 0,079 | 1,104 | 1,352 | 0,000 | 0,000 |
| Bacteroidia | Bacteroidales | Bacteroidaceae | Bacteroides | vulgatus | 0,378 | 0,461 | 0,132 | 0,731 | 0,800 | 0,000 | 0,000 |
| Bacteroidia | Bacteroidales | Bacteroidales_S24-7_group | NA | NA | 0,035 | 0,028 | 0,001 | 3,494 | 3,318 | 0,000 | 0,000 |
| Bacteroidia | Bacteroidales | Bacteroidales_S24-7_group | NA | NA | 0,008 | 0,073 | 0,001 | 2,387 | 4,226 | 0,000 | 0,000 |
| Bacteroidia | Bacteroidales | M2PB4-65_termite_group | NA | NA | 0,019 | 0,025 | 0,000 | 3,015 | 4,025 | 0,001 | 0,000 |
| Bacteroidia | Bacteroidales | Porphyromonadaceae | Barnesiella | NA | 0,030 | 0,058 | 0,007 | 1,777 | 2,452 | 0,058 | 0,005 |
| Bacteroidia | Bacteroidales | Porphyromonadaceae | Coprobacter | NA | 0,016 | 0,023 | 0,001 | 3,125 | 3,738 | 0,000 | 0,000 |
| Bacteroidia | Bacteroidales | Porphyromonadaceae | Dysgonomonas | oryzarvi | 0,060 | 0,044 | 0,012 | 2,477 | 2,155 | 0,000 | 0,000 |
| Bacteroidia | Bacteroidales | Porphyromonadaceae | Macellibacteroides | fermentans | 0,270 | 0,309 | 0,218 | 0,386 | 0,386 | 0,070 | 0,070 |
| Bacteroidia | Bacteroidales | Porphyromonadaceae | Macellibacteroides | NA | 0,012 | 0,074 | 0,002 | 2,250 | 4,121 | 0,004 | 0,000 |
| Bacteroidia | Bacteroidales | Porphyromonadaceae | Microbacter | NA | 0,031 | 0,058 | 0,001 | 3,654 | 4,233 | 0,000 | 0,000 |
| Bacteroidia | Bacteroidales | Porphyromonadaceae | Microbacter | NA | 0,009 | 0,044 | 0,000 | 3,259 | 4,248 | 0,000 | 0,000 |
| Bacteroidia | Bacteroidales | Porphyromonadaceae | NA | NA | 0,011 | 0,015 | 0,000 | 3,126 | 3,494 | 0,000 | 0,000 |
| Bacteroidia | Bacteroidales | Porphyromonadaceae | NA | NA | 0,058 | 0,052 | 0,018 | 1,086 | 1,254 | 0,014 | 0,003 |
| Bacteroidia | Bacteroidales | Porphyromonadaceae | Odoribacter | splanchnicus | 0,032 | 0,056 | 0,006 | 2,014 | 2,556 | 0,000 | 0,000 |
| Bacteroidia | Bacteroidales | Porphyromonadaceae | Odoribacter | splanchnicus | 0,044 | 0,058 | 0,007 | 1,796 | 2,299 | 0,001 | 0,000 |
| Bacteroidia | Bacteroidales | Porphyromonadaceae | Paludibacter | NA | 0,023 | 0,020 | 0,000 | 3,526 | 3,760 | 0,000 | 0,000 |
| Bacteroidia | Bacteroidales | Porphyromonadaceae | Paludibacter | NA | 0,104 | 0,193 | 0,008 | 2,689 | 3,051 | 0,000 | 0,000 |
| Bacteroidia | Bacteroidales | Porphyromonadaceae | Paludibacter | NA | 0,010 | 0,158 | 0,002 | 1,696 | 4,253 | 0,054 | 0,000 |
| Bacteroidia | Bacteroidales | Porphyromonadaceae | Paludibacter | NA | 0,166 | 0,067 | 0,034 | 1,276 | 0,809 | 0,000 | 0,025 |
| Bacteroidia | Bacteroidales | Porphyromonadaceae | Paludibacter | NA | 0,164 | 0,345 | 0,024 | 1,223 | 1,737 | 0,000 | 0,000 |
| Bacteroidia | Bacteroidales | Porphyromonadaceae | Parabacteroides | distasonis | 0,081 | 0,109 | 0,022 | 1,943 | 2,098 | 0,000 | 0,000 |
| Bacteroidia | Bacteroidales | Porphyromonadaceae | Parabacteroides | distasonis | 0,021 | 0,034 | 0,007 | 1,801 | 2,061 | 0,002 | 0,000 |
| Bacteroidia | Bacteroidales | Porphyromonadaceae | Parabacteroides | goldsteinii | 0,011 | 0,060 | 0,000 | 2,797 | 4,477 | 0,000 | 0,000 |
| Bacteroidia | Bacteroidales | Porphyromonadaceae | Parabacteroides | merdae | 0,161 | 0,224 | 0,114 | 0,427 | 0,560 | 0,064 | 0,009 |
| Bacteroidia | Bacteroidales | Porphyromonadaceae | Parabacteroides | NA | 0,080 | 0,011 | 0,002 | 3,942 | 2,156 | 0,000 | 0,004 |
| Bacteroidia | Bacteroidales | Porphyromonadaceae | Proteiniphilum | NA | 0,003 | 0,016 | 0,000 | 1,889 | 3,716 | 0,059 | 0,000 |
| Bacteroidia | Bacteroidales | Prevotellaceae | Paraprevotella | NA | 0,024 | 0,050 | 0,001 | 3,231 | 4,130 | 0,000 | 0,000 |
| Bacteroidia | Bacteroidales | Prevotellaceae | Paraprevotella | NA | 0,033 | 0,019 | 0,004 | 2,813 | 2,465 | 0,000 | 0,000 |
| Bacteroidia | Bacteroidales | Prevotellaceae | Prevotella_9 | NA | 1,061 | 0,938 | 0,011 | 4,128 | 4,223 | 0,000 | 0,000 |
| Bacteroidia | Bacteroidales | Prevotellaceae | Prevotella_9 | NA | 0,023 | 0,029 | 0,000 | 2,969 | 3,577 | 0,028 | 0,005 |
| Bacteroidia | Bacteroidales | Prevotellaceae | Prevotella_9 | NA | 0,076 | 0,104 | 0,018 | 1,848 | 2,003 | 0,013 | 0,006 |
| Bacteroidia | Bacteroidales | Prevotellaceae | Prevotella_9 | NA | 0,008 | 0,130 | 0,002 | 1,755 | 4,406 | 0,026 | 0,000 |
| Bacteroidia | Bacteroidales | Prevotellaceae | Prevotellaceae_NK3B31_group | NA | 0,029 | 0,019 | 0,001 | 3,479 | 2,740 | 0,001 | 0,015 |
| Bacteroidia | Bacteroidales | Prolixibacteraceae | Prolixibacter | NA | 0,021 | 0,014 | 0,000 | 3,611 | 3,291 | 0,000 | 0,000 |
| Bacteroidia | Bacteroidales | Prolixibacteraceae | Prolixibacter | NA | 0,010 | 0,009 | 0,000 | 2,554 | 2,410 | 0,045 | 0,062 |
| Bacteroidia | Bacteroidales | Rikenellaceae | Alistipes | finegoldii | 0,031 | 0,048 | 0,000 | 3,971 | 4,480 | 0,000 | 0,000 |
| Bacteroidia | Bacteroidales | Rikenellaceae | Alistipes | inops | 0,030 | 0,034 | 0,007 | 1,815 | 2,075 | 0,000 | 0,000 |
| Bacteroidia | Bacteroidales | Rikenellaceae | Alistipes | NA | 0,046 | 0,018 | 0,002 | 3,258 | 2,322 | 0,000 | 0,003 |
| Bacteroidia | Bacteroidales | Rikenellaceae | Alistipes | NA | 0,005 | 0,005 | 0,000 | 2,445 | 2,546 | 0,096 | 0,082 |
| Bacteroidia | Bacteroidales | Rikenellaceae | Alistipes | NA | 0,015 | 0,014 | 0,003 | 2,414 | 2,317 | 0,024 | 0,032 |
| Bacteroidia | Bacteroidales | Rikenellaceae | Alistipes | NA | 0,129 | 0,169 | 0,015 | 1,861 | 2,109 | 0,000 | 0,000 |
| Bacteroidia | Bacteroidales | Rikenellaceae | Alistipes | NA | 0,014 | 0,011 | 0,004 | 1,791 | 1,893 | 0,023 | 0,014 |
| Bacteroidia | Bacteroidales | Rikenellaceae | Alistipes | obesi | 0,024 | 0,024 | 0,004 | 2,062 | 2,372 | 0,000 | 0,000 |
| Bacteroidia | Bacteroidales | Rikenellaceae | Alistipes | putredinis | 0,258 | 0,251 | 0,040 | 1,071 | 1,131 | 0,000 | 0,000 |
| Bacteroidia | Bacteroidales | Rikenellaceae | Alistipes | shahii | 0,035 | 0,043 | 0,000 | 3,757 | 4,095 | 0,000 | 0,000 |
| Bacteroidia | Bacteroidales | Rikenellaceae | Anaerocella | delicata | 0,101 | 0,057 | 0,001 | 4,270 | 4,090 | 0,000 | 0,000 |
| Bacteroidia | Bacteroidales | Rikenellaceae | dgA-11_gut_group | NA | 0,392 | 0,017 | 0,008 | 2,790 | 1,366 | 0,000 | 0,001 |
| Bacteroidia | Bacteroidales | Rikenellaceae | vadinBC27_wastewater-sludge_group | NA | 0,057 | 0,039 | 0,007 | 2,162 | 2,013 | 0,000 | 0,000 |
| Bacteroidia | Bacteroidales | Rikenellaceae | vadinBC27_wastewater-sludge_group | NA | 0,016 | 0,024 | 0,007 | 1,281 | 1,772 | 0,022 | 0,001 |
| Bacteroidia | Bacteroidales | Rikenellaceae | vadinBC27_wastewater-sludge_group | NA | 0,099 | 0,039 | 0,025 | 0,835 | 0,608 | 0,004 | 0,055 |
| Flavobacteriia | Flavobacteriales | Flavobacteriaceae | Cloacibacterium | normanense | 1,412 | 0,542 | 0,339 | 0,625 | 0,337 | 0,000 | 0,098 |
| Flavobacteriia | Flavobacteriales | Flavobacteriaceae | Flavobacterium | NA | 0,015 | 0,041 | 0,002 | 2,755 | 3,668 | 0,000 | 0,000 |
| Flavobacteriia | Flavobacteriales | Flavobacteriaceae | Flavobacterium | NA | 0,141 | 0,092 | 0,020 | 1,817 | 1,688 | 0,000 | 0,000 |
| Sphingobacteriia | Sphingobacteriales | KD1-131 | NA | NA | 0,004 | 0,018 | 0,000 | 1,888 | 3,825 | 0,045 | 0,000 |
| Sphingobacteriia | Sphingobacteriales | ST-12K33 | NA | NA | 0,041 | 0,017 | 0,004 | 2,753 | 2,102 | 0,005 | 0,045 |
| Sphingobacteriia | Sphingobacteriales | ST-12K33 | NA | NA | 0,096 | 0,033 | 0,036 | 1,479 | 0,722 | 0,000 | 0,068 |
| Chlorobia | Chlorobiales | OPB56 | NA | NA | 0,022 | 0,015 | 0,001 | 2,792 | 3,194 | 0,001 | 0,000 |
| Melainabacteria | Gastranaerophilales | NA | NA | NA | 0,008 | 0,022 | 0,002 | 1,580 | 2,779 | 0,073 | 0,000 |
| Fibrobacteria | Fibrobacterales | Fibrobacteraceae | possible_genus_06 | NA | 0,055 | 0,030 | 0,001 | 4,098 | 3,612 | 0,000 | 0,000 |
| Fibrobacteria | Fibrobacterales | Fibrobacteraceae | possible_genus_06 | NA | 0,044 | 0,006 | 0,002 | 3,651 | 2,020 | 0,000 | 0,030 |
| Bacilli | Lactobacillales | Lactobacillaceae | Lactobacillus | delbrueckii | 0,889 | 0,041 | 0,002 | 4,867 | 2,540 | 0,000 | 0,000 |
| Bacilli | Lactobacillales | Lactobacillaceae | Lactobacillus | delbrueckii | 1,278 | 0,301 | 0,013 | 4,189 | 3,898 | 0,000 | 0,000 |
| Bacilli | Lactobacillales | Lactobacillaceae | Lactobacillus | NA | 0,096 | 0,040 | 0,001 | 3,822 | 4,070 | 0,000 | 0,000 |
| Bacilli | Lactobacillales | Lactobacillaceae | Lactobacillus | NA | 0,005 | 0,029 | 0,001 | 1,565 | 3,652 | 0,051 | 0,000 |
| Bacilli | Lactobacillales | Streptococcaceae | Lactococcus | NA | 0,492 | 3,768 | 0,074 | 0,921 | 1,442 | 0,000 | 0,000 |
| Bacilli | Lactobacillales | Streptococcaceae | Lactococcus | NA | 0,269 | 0,970 | 0,039 | 0,920 | 1,443 | 0,000 | 0,000 |
| Bacilli | Lactobacillales | Streptococcaceae | Lactococcus | raffinolactis | 0,321 | 1,994 | 0,079 | 1,448 | 2,043 | 0,000 | 0,000 |
| Bacilli | Lactobacillales | Streptococcaceae | Streptococcus | NA | 1,384 | 0,219 | 0,067 | 1,147 | 0,448 | 0,000 | 0,062 |
| Clostridia | Clostridiales | Christensenellaceae | Christensenellaceae_R-7_group | NA | 0,068 | 0,010 | 0,000 | 4,445 | 2,646 | 0,000 | 0,009 |
| Clostridia | Clostridiales | Christensenellaceae | Christensenellaceae_R-7_group | NA | 0,010 | 0,019 | 0,002 | 2,693 | 3,253 | 0,000 | 0,000 |
| Clostridia | Clostridiales | Christensenellaceae | Christensenellaceae_R-7_group | NA | 0,041 | 0,079 | 0,005 | 2,327 | 2,810 | 0,000 | 0,000 |
| Clostridia | Clostridiales | Christensenellaceae | Christensenellaceae_R-7_group | NA | 0,089 | 0,047 | 0,021 | 1,293 | 1,112 | 0,000 | 0,000 |
| Clostridia | Clostridiales | Christensenellaceae | Christensenellaceae_R-7_group | NA | 0,021 | 0,047 | 0,015 | 0,612 | 1,220 | 0,084 | 0,000 |
| Clostridia | Clostridiales | Clostridiaceae_1 | Proteiniclasticum | ruminis | 0,083 | 0,096 | 0,005 | 3,239 | 3,354 | 0,000 | 0,000 |
| Clostridia | Clostridiales | Eubacteriaceae | Anaerofustis | NA | 0,010 | 0,007 | 0,002 | 2,379 | 1,944 | 0,002 | 0,017 |
| Clostridia | Clostridiales | Eubacteriaceae | NA | NA | 3,518 | 0,147 | 0,049 | 1,778 | 0,840 | 0,000 | 0,000 |
| Clostridia | Clostridiales | Family_XI | NA | NA | 0,020 | 0,040 | 0,010 | 1,159 | 1,914 | 0,010 | 0,000 |
| Clostridia | Clostridiales | Family_XI | Tissierella | NA | 0,025 | 0,009 | 0,000 | 3,992 | 3,131 | 0,000 | 0,000 |
| Clostridia | Clostridiales | Family_XI | Tissierella | NA | 0,013 | 0,045 | 0,001 | 2,442 | 3,754 | 0,000 | 0,000 |
| Clostridia | Clostridiales | Lachnospiraceae | Butyrivibrio | crossotus | 0,027 | 0,050 | 0,005 | 2,227 | 2,673 | 0,000 | 0,000 |
| Clostridia | Clostridiales | Lachnospiraceae | Coprococcus_2 | eutactus | 0,057 | 0,103 | 0,005 | 2,402 | 3,113 | 0,000 | 0,000 |
| Clostridia | Clostridiales | Lachnospiraceae | Coprococcus_2 | NA | 0,041 | 0,010 | 0,001 | 3,899 | 2,524 | 0,000 | 0,000 |
| Clostridia | Clostridiales | Lachnospiraceae | Fusicatenibacter | NA | 0,034 | 0,046 | 0,013 | 1,333 | 1,603 | 0,000 | 0,000 |
| Clostridia | Clostridiales | Lachnospiraceae | Howardella | NA | 0,003 | 0,010 | 0,002 | 1,545 | 2,994 | 0,080 | 0,000 |
| Clostridia | Clostridiales | Lachnospiraceae | NA | NA | 0,098 | 0,049 | 0,000 | 4,898 | 4,200 | 0,000 | 0,000 |
| Clostridia | Clostridiales | Lachnospiraceae | NA | NA | 0,029 | 0,046 | 0,003 | 2,641 | 2,965 | 0,000 | 0,000 |
| Clostridia | Clostridiales | Lachnospiraceae | NA | NA | 0,008 | 0,007 | 0,002 | 2,295 | 2,076 | 0,016 | 0,032 |
| Clostridia | Clostridiales | Lachnospiraceae | NA | NA | 0,047 | 0,101 | 0,015 | 1,246 | 1,736 | 0,000 | 0,000 |
| Clostridia | Clostridiales | Lachnospiraceae | NA | NA | 0,018 | 0,027 | 0,009 | 1,039 | 1,475 | 0,093 | 0,009 |
| Clostridia | Clostridiales | Lachnospiraceae | NA | NA | 0,196 | 0,132 | 0,055 | 0,911 | 0,729 | 0,000 | 0,006 |
| Clostridia | Clostridiales | Lachnospiraceae | Roseburia | intestinalis | 0,031 | 0,024 | 0,004 | 2,928 | 2,630 | 0,016 | 0,033 |
| Clostridia | Clostridiales | Lachnospiraceae | Roseburia | NA | 0,020 | 0,106 | 0,011 | 0,909 | 2,152 | 0,079 | 0,000 |
| Clostridia | Clostridiales | Ruminococcaceae | Ercella | NA | 0,023 | 0,030 | 0,004 | 2,589 | 2,803 | 0,000 | 0,000 |
| Clostridia | Clostridiales | Ruminococcaceae | Intestinimonas | NA | 0,009 | 0,012 | 0,000 | 2,927 | 3,134 | 0,022 | 0,012 |
| Clostridia | Clostridiales | Ruminococcaceae | Intestinimonas | NA | 0,011 | 0,019 | 0,000 | 2,713 | 3,848 | 0,006 | 0,000 |
| Clostridia | Clostridiales | Ruminococcaceae | Intestinimonas | NA | 0,007 | 0,018 | 0,001 | 2,059 | 3,405 | 0,023 | 0,000 |
| Clostridia | Clostridiales | Ruminococcaceae | NA | NA | 0,015 | 0,057 | 0,000 | 3,659 | 4,585 | 0,000 | 0,000 |
| Clostridia | Clostridiales | Ruminococcaceae | NA | NA | 0,010 | 0,020 | 0,000 | 2,720 | 3,783 | 0,001 | 0,000 |
| Clostridia | Clostridiales | Ruminococcaceae | NA | NA | 0,005 | 0,012 | 0,000 | 2,259 | 3,324 | 0,070 | 0,003 |
| Clostridia | Clostridiales | Ruminococcaceae | NA | NA | 0,023 | 0,035 | 0,010 | 1,111 | 1,662 | 0,009 | 0,000 |
| Clostridia | Clostridiales | Ruminococcaceae | NA | NA | 0,024 | 0,020 | 0,010 | 0,987 | 0,965 | 0,057 | 0,064 |
| Clostridia | Clostridiales | Ruminococcaceae | NA | NA | 0,067 | 0,112 | 0,039 | 0,578 | 0,793 | 0,025 | 0,001 |
| Clostridia | Clostridiales | Ruminococcaceae | Ruminiclostridium_5 | NA | 0,016 | 0,048 | 0,004 | 1,937 | 2,978 | 0,000 | 0,000 |
| Clostridia | Clostridiales | Ruminococcaceae | Ruminiclostridium_6 | NA | 0,043 | 0,102 | 0,001 | 4,025 | 4,572 | 0,000 | 0,000 |
| Clostridia | Clostridiales | Ruminococcaceae | Ruminococcaceae_NK4A214_group | NA | 0,020 | 0,014 | 0,004 | 2,291 | 1,850 | 0,001 | 0,011 |
| Clostridia | Clostridiales | Ruminococcaceae | Ruminococcaceae_UCG-002 | NA | 0,006 | 0,033 | 0,000 | 1,969 | 4,062 | 0,093 | 0,000 |
| Clostridia | Clostridiales | Ruminococcaceae | Ruminococcaceae_UCG-002 | NA | 0,097 | 0,112 | 0,047 | 0,584 | 0,731 | 0,021 | 0,002 |
| Clostridia | Clostridiales | Ruminococcaceae | Ruminococcaceae_UCG-002 | NA | 0,117 | 0,160 | 0,103 | 0,405 | 0,543 | 0,096 | 0,016 |
| Clostridia | Clostridiales | Ruminococcaceae | Ruminococcaceae_UCG-004 | NA | 0,008 | 0,020 | 0,004 | 1,294 | 2,608 | 0,060 | 0,000 |
| Clostridia | Clostridiales | Ruminococcaceae | Ruminococcaceae_UCG-005 | NA | 0,013 | 0,018 | 0,000 | 2,764 | 3,447 | 0,010 | 0,001 |
| Clostridia | Clostridiales | Ruminococcaceae | Ruminococcaceae_UCG-010 | NA | 0,005 | 0,006 | 0,002 | 2,084 | 2,360 | 0,015 | 0,004 |
| Clostridia | Clostridiales | Ruminococcaceae | Ruminococcaceae_UCG-014 | NA | 0,035 | 0,117 | 0,008 | 2,031 | 2,554 | 0,000 | 0,000 |
| Clostridia | Clostridiales | Ruminococcaceae | Ruminococcaceae_UCG-014 | NA | 0,046 | 0,047 | 0,026 | 0,689 | 0,562 | 0,019 | 0,069 |
| Clostridia | Clostridiales | Ruminococcaceae | Ruminococcus_1 | bicirculans | 0,086 | 0,073 | 0,002 | 3,944 | 3,830 | 0,000 | 0,000 |
| Clostridia | Clostridiales | Ruminococcaceae | Ruminococcus_1 | NA | 0,009 | 0,012 | 0,000 | 2,970 | 3,202 | 0,010 | 0,004 |
| Clostridia | Clostridiales | Ruminococcaceae | Ruminococcus_1 | NA | 0,004 | 0,018 | 0,000 | 2,045 | 3,988 | 0,017 | 0,000 |
| Clostridia | Clostridiales | Ruminococcaceae | Ruminococcus_2 | bromii | 0,084 | 0,150 | 0,023 | 1,259 | 1,777 | 0,000 | 0,000 |
| Clostridia | Clostridiales | Ruminococcaceae | Ruminococcus_2 | bromii | 0,183 | 0,126 | 0,081 | 0,806 | 0,570 | 0,000 | 0,020 |
| Clostridia | Clostridiales | Ruminococcaceae | Ruminococcus_2 | NA | 0,110 | 0,071 | 0,031 | 1,124 | 1,038 | 0,001 | 0,002 |
| Erysipelotrichia | Erysipelotrichales | Erysipelotrichaceae | Catenisphaera | NA | 0,018 | 0,024 | 0,007 | 1,525 | 2,066 | 0,023 | 0,001 |
| Erysipelotrichia | Erysipelotrichales | Erysipelotrichaceae | Erysipelothrix | NA | 0,045 | 0,018 | 0,004 | 3,035 | 1,753 | 0,000 | 0,032 |
| Erysipelotrichia | Erysipelotrichales | Erysipelotrichaceae | Erysipelothrix | NA | 0,008 | 0,009 | 0,001 | 2,553 | 2,756 | 0,002 | 0,001 |
| Erysipelotrichia | Erysipelotrichales | Erysipelotrichaceae | Faecalitalea | cylindroides | 0,005 | 0,015 | 0,003 | 1,676 | 2,694 | 0,045 | 0,000 |
| Erysipelotrichia | Erysipelotrichales | Erysipelotrichaceae | Faecalitalea | NA | 0,005 | 0,009 | 0,000 | 2,326 | 2,984 | 0,097 | 0,023 |
| Erysipelotrichia | Erysipelotrichales | Erysipelotrichaceae | NA | NA | 0,009 | 0,013 | 0,001 | 2,548 | 2,847 | 0,037 | 0,016 |
| Negativicutes | Selenomonadales | Acidaminococcaceae | Phascolarctobacterium | NA | 0,028 | 0,019 | 0,007 | 2,123 | 1,620 | 0,001 | 0,014 |
| Negativicutes | Selenomonadales | Veillonellaceae | Anaeroarcus | burkinensis | 0,083 | 0,071 | 0,004 | 3,073 | 2,925 | 0,000 | 0,000 |
| Negativicutes | Selenomonadales | Veillonellaceae | Anaeroarcus | NA | 0,237 | 0,204 | 0,036 | 1,090 | 0,995 | 0,000 | 0,000 |
| Negativicutes | Selenomonadales | Veillonellaceae | Megasphaera | NA | 0,126 | 0,040 | 0,001 | 4,217 | 3,448 | 0,000 | 0,000 |
| Negativicutes | Selenomonadales | Veillonellaceae | NA | NA | 0,043 | 0,046 | 0,023 | 0,868 | 1,039 | 0,005 | 0,000 |
| Negativicutes | Selenomonadales | Veillonellaceae | Selenomonas | lacticifex | 0,070 | 0,120 | 0,006 | 2,733 | 2,996 | 0,000 | 0,000 |
| Negativicutes | Selenomonadales | Veillonellaceae | Veillonella | NA | 0,250 | 0,287 | 0,001 | 4,295 | 4,773 | 0,000 | 0,000 |
| Fusobacteriia | Fusobacteriales | Leptotrichiaceae | NA | NA | 0,303 | 0,047 | 0,004 | 4,138 | 3,259 | 0,000 | 0,000 |
| Bacteria beloning tot he phylum Gracilibacteria | | | | | 0,078 | 0,037 | 0,008 | 2,052 | 1,806 | 0,000 | 0,000 |
| Lentisphaeria | Victivallales | Victivallaceae | Victivallis | NA | 0,010 | 0,012 | 0,004 | 1,337 | 1,797 | 0,095 | 0,015 |
| Alphaproteobacteria | Rhodospirillales | Rhodospirillaceae | Elstera | NA | 0,018 | 0,007 | 0,002 | 3,026 | 2,273 | 0,000 | 0,009 |
| Alphaproteobacteria | Rhodospirillales | Rhodospirillaceae | NA | NA | 0,014 | 0,014 | 0,004 | 1,621 | 1,237 | 0,017 | 0,088 |
| Alphaproteobacteria | Rhodospirillales | Rhodospirillaceae | NA | NA | 0,045 | 0,045 | 0,020 | 1,023 | 1,129 | 0,003 | 0,001 |
| Betaproteobacteria | Burkholderiales | Alcaligenaceae | Sutterella | NA | 0,006 | 0,046 | 0,000 | 2,555 | 4,339 | 0,004 | 0,000 |
| Betaproteobacteria | Burkholderiales | Comamonadaceae | Acidovorax | NA | 0,032 | 0,044 | 0,007 | 2,462 | 2,693 | 0,001 | 0,000 |
| Betaproteobacteria | Burkholderiales | Comamonadaceae | Diaphorobacter | NA | 0,163 | 0,075 | 0,039 | 1,540 | 1,125 | 0,000 | 0,000 |
| Betaproteobacteria | DR-16 | NA | NA | NA | 0,684 | 0,093 | 0,000 | 5,237 | 4,495 | 0,000 | 0,000 |
| Betaproteobacteria | Neisseriales | Neisseriaceae | Formivibrio | NA | 0,026 | 0,149 | 0,003 | 2,521 | 3,433 | 0,000 | 0,000 |
| Betaproteobacteria | Neisseriales | Neisseriaceae | Laribacter | hongkongensis | 0,081 | 0,035 | 0,019 | 2,057 | 1,437 | 0,000 | 0,000 |
| Betaproteobacteria | Neisseriales | Neisseriaceae | Microvirgula | aerodenitrificans | 0,012 | 0,040 | 0,001 | 3,069 | 3,871 | 0,000 | 0,000 |
| Betaproteobacteria | Rhodocyclales | Rhodocyclaceae | Dechlorobacter | NA | 0,262 | 0,212 | 0,106 | 1,164 | 0,986 | 0,000 | 0,000 |
| Betaproteobacteria | Rhodocyclales | Rhodocyclaceae | Dechloromonas | NA | 0,511 | 0,168 | 0,032 | 1,671 | 1,388 | 0,000 | 0,000 |
| Betaproteobacteria | Rhodocyclales | Rhodocyclaceae | Propionivibrio | NA | 0,061 | 0,017 | 0,000 | 4,357 | 3,322 | 0,000 | 0,000 |
| Betaproteobacteria | Rhodocyclales | Rhodocyclaceae | Propionivibrio | NA | 0,345 | 0,090 | 0,053 | 1,513 | 0,956 | 0,000 | 0,000 |
| Deltaproteobacteria | Desulfobacterales | Desulfobulbaceae | Desulfobulbus | NA | 0,032 | 0,006 | 0,000 | 3,995 | 2,380 | 0,000 | 0,033 |
| Deltaproteobacteria | Desulfovibrionales | Desulfovibrionaceae | Desulfovibrio | desulfuricans | 0,205 | 0,099 | 0,086 | 0,728 | 0,426 | 0,001 | 0,094 |
| Deltaproteobacteria | Desulfovibrionales | Desulfovibrionaceae | Desulfovibrio | intestinalis | 0,058 | 0,067 | 0,022 | 1,258 | 1,123 | 0,000 | 0,000 |
| Epsilonproteobacteria | Campylobacterales | Campylobacteraceae | Arcobacter | aquimarinus | 0,139 | 0,413 | 0,025 | 1,548 | 2,366 | 0,006 | 0,000 |
| Epsilonproteobacteria | Campylobacterales | Campylobacteraceae | Arcobacter | butzleri | 0,795 | 0,769 | 0,183 | 0,952 | 1,251 | 0,000 | 0,000 |
| Epsilonproteobacteria | Campylobacterales | Campylobacteraceae | Arcobacter | cryaerophilus | 11,237 | 10,135 | 4,013 | 0,363 | 0,355 | 0,017 | 0,019 |
| Epsilonproteobacteria | Campylobacterales | Campylobacteraceae | Arcobacter | NA | 0,564 | 0,382 | 0,118 | 1,964 | 1,931 | 0,000 | 0,000 |
| Epsilonproteobacteria | Campylobacterales | Campylobacteraceae | Arcobacter | NA | 0,115 | 0,285 | 0,036 | 1,803 | 2,728 | 0,021 | 0,000 |
| Epsilonproteobacteria | Campylobacterales | Campylobacteraceae | Arcobacter | NA | 0,035 | 0,129 | 0,008 | 1,514 | 2,945 | 0,068 | 0,000 |
| Epsilonproteobacteria | Campylobacterales | Campylobacteraceae | Sulfurospirillum | cavolei | 0,005 | 0,090 | 0,000 | 2,384 | 4,425 | 0,017 | 0,000 |
| Epsilonproteobacteria | Campylobacterales | Campylobacteraceae | Sulfurospirillum | NA | 0,009 | 0,119 | 0,003 | 1,590 | 3,704 | 0,010 | 0,000 |
| Gammaproteobacteria | Aeromonadales | Aeromonadaceae | Aeromonas | NA | 0,257 | 0,007 | 0,000 | 5,114 | 1,647 | 0,000 | 0,038 |
| Gammaproteobacteria | Aeromonadales | Aeromonadaceae | Tolumonas | NA | 0,183 | 0,494 | 0,038 | 1,744 | 2,394 | 0,000 | 0,000 |
| Gammaproteobacteria | Aeromonadales | Aeromonadaceae | Tolumonas | NA | 0,007 | 0,180 | 0,002 | 1,331 | 4,702 | 0,093 | 0,000 |
| Gammaproteobacteria | Enterobacteriales | Enterobacteriaceae | Citrobacter | NA | 0,024 | 0,031 | 0,004 | 2,510 | 3,010 | 0,015 | 0,002 |
| Gammaproteobacteria | Enterobacteriales | Enterobacteriaceae | Enterobacter | NA | 0,325 | 0,088 | 0,040 | 0,569 | 0,507 | 0,048 | 0,086 |
| Gammaproteobacteria | Enterobacteriales | Enterobacteriaceae | Klebsiella | NA | 0,159 | 0,021 | 0,010 | 2,686 | 1,283 | 0,000 | 0,018 |
| Gammaproteobacteria | Pseudomonadales | Moraxellaceae | Acinetobacter | bouvetii | 0,052 | 0,154 | 0,017 | 1,641 | 1,597 | 0,080 | 0,091 |
| Gammaproteobacteria | Pseudomonadales | Moraxellaceae | Acinetobacter | haemolyticus | 0,024 | 0,152 | 0,002 | 2,857 | 4,262 | 0,000 | 0,000 |
| Gammaproteobacteria | Pseudomonadales | Moraxellaceae | Acinetobacter | johnsonii | 0,166 | 0,184 | 0,004 | 4,104 | 3,683 | 0,000 | 0,000 |
| Gammaproteobacteria | Pseudomonadales | Moraxellaceae | Acinetobacter | johnsonii | 0,408 | 0,520 | 0,147 | 0,396 | 0,543 | 0,065 | 0,006 |
| Gammaproteobacteria | Pseudomonadales | Moraxellaceae | Acinetobacter | NA | 0,046 | 0,160 | 0,000 | 3,475 | 4,716 | 0,000 | 0,000 |
| Gammaproteobacteria | Pseudomonadales | Moraxellaceae | Acinetobacter | NA | 0,014 | 0,428 | 0,003 | 1,706 | 4,335 | 0,006 | 0,000 |
| Gammaproteobacteria | Pseudomonadales | Moraxellaceae | Acinetobacter | NA | 0,567 | 0,924 | 0,187 | 0,448 | 0,601 | 0,026 | 0,001 |
| Gammaproteobacteria | Pseudomonadales | Pseudomonadaceae | Pseudomonas | NA | 0,024 | 0,066 | 0,008 | 1,644 | 2,285 | 0,000 | 0,000 |
| Gammaproteobacteria | Pseudomonadales | Pseudomonadaceae | Pseudomonas | NA | 0,128 | 0,046 | 0,035 | 1,440 | 0,721 | 0,000 | 0,031 |
| Gammaproteobacteria | Pseudomonadales | Pseudomonadaceae | Pseudomonas | pseudoalcaligenes | 1,274 | 0,159 | 0,110 | 1,143 | 0,619 | 0,000 | 0,005 |
| Gammaproteobacteria | Xanthomonadales | Xanthomonadaceae | Stenotrophomonas | maltophilia | 0,018 | 0,031 | 0,006 | 2,004 | 2,344 | 0,093 | 0,042 |
| Bacteria belonging to the phylum Saccharibacteria | | | | | 0,013 | 0,021 | 0,000 | 3,413 | 3,467 | 0,001 | 0,000 |
| Bacteria belonging to the phylum Saccharibacteria | | | | | 0,009 | 0,052 | 0,000 | 3,087 | 4,397 | 0,000 | 0,000 |
| Bacteria belonging to the phylum Saccharibacteria | | | | | 0,028 | 0,078 | 0,005 | 2,116 | 2,783 | 0,026 | 0,002 |
| Bacteria belonging to the phylum Saccharibacteria | | | | | 0,064 | 0,068 | 0,018 | 1,075 | 1,172 | 0,000 | 0,000 |
| Synergistia | Synergistales | Synergistaceae | Lactivibrio | NA | 0,061 | 0,004 | 0,001 | 3,897 | 1,760 | 0,000 | 0,056 |
| Mollicutes | NB1-n | NA | NA | NA | 0,005 | 0,013 | 0,000 | 2,051 | 3,759 | 0,026 | 0,000 |
| Verrucomicrobia OPB35_soil_group | NA | NA | NA | NA | 0,167 | 0,013 | 0,000 | 5,010 | 3,761 | 0,000 | 0,000 |

**Table S4. Clinical indicator bacteria in influent.** 10 of the 207 *clinical enriched taxa* were found to be significant more abundant in influent (I) when compared to community (C) wastewater. 4 of the *clinical indicator bacteria* were found to be more abundant in community wastewater when compared to influent (shown in grey), however, for three species the difference was not significant (p > 0.05).

| **Bacterial identification** | | | | | | | **Mean abundance (%)** | | **Log2-FoldChange** | **p-value** |
| --- | --- | --- | --- | --- | --- | --- | --- | --- | --- | --- |
| **Kingdom** | **Phylum** | **Class** | **Order** | **Family** | **Genus** | **Species** | **I** | **C** | **I - C** |  |
| Bacteria | Proteobacteria | Gammaproteobacteria | Aeromonadales | Aeromonadaceae | Tolumonas | NA | 0,198 | 0,038 | 0,780 | 0,008 |
| Bacteria | Bacteroidetes | Bacteroidia | Bacteroidales | Prevotellaceae | Prevotella_9 | NA | 0,153 | 0,018 | 0,667 | 0,040 |
| Bacteria | Proteobacteria | Deltaproteobacteria | Desulfovibrionales | Desulfovibrionaceae | Desulfovibrio | desulfuricans | 0,038 | 0,086 | -0,585 | 0,036 |
| Bacteria | Proteobacteria | Epsilonproteobacteria | Campylobacterales | Campylobacteraceae | Arcobacter | NA | 1,940 | 0,036 | 1,600 | 0,000 |
| Bacteria | Bacteroidetes | Bacteroidia | Bacteroidales | Porphyromonadaceae | Paludibacter | NA | 0,126 | 0,002 | 0,982 | 0,006 |
| Bacteria | Firmicutes | Negativicutes | Selenomonadales | Veillonellaceae | Selenomonas | lacticifex | 0,120 | 0,006 | 0,832 | 0,007 |
| Bacteria | Bacteroidetes | Flavobacteriia | Flavobacteriales | Flavobacteriaceae | Cloacibacterium | normanense | 0,272 | 0,339 | -0,438 | 0,099 |
| Bacteria | Proteobacteria | Gammaproteobacteria | Aeromonadales | Aeromonadaceae | Tolumonas | NA | 0,115 | 0,002 | 0,980 | 0,007 |
| Bacteria | Bacteroidetes | Bacteroidia | Bacteroidales | Bacteroidaceae | Bacteroides | stercoris | 0,074 | 0,002 | 0,835 | 0,036 |
| Bacteria | Bacteroidetes | Bacteroidia | Bacteroidales | Prevotellaceae | Prevotella_9 | NA | 0,093 | 0,011 | 0,818 | 0,017 |
| Bacteria | Firmicutes | Bacilli | Lactobacillales | Lactobacillaceae | Lactobacillus | delbrueckii | 0,112 | 0,013 | 0,940 | 0,005 |
| Bacteria | Proteobacteria | Epsilonproteobacteria | Campylobacterales | Campylobacteraceae | Arcobacter | aquimarinus | 0,759 | 0,025 | 1,173 | 0,000 |
| Bacteria | Proteobacteria | Gammaproteobacteria | Pseudomonadales | Pseudomonadaceae | Pseudomonas | pseudoalcaligenes | 0,056 | 0,110 | -0,576 | 0,059 |
| Bacteria | Proteobacteria | Deltaproteobacteria | Desulfovibrionales | Desulfovibrionaceae | Desulfovibrio | intestinalis | 0,010 | 0,022 | -0,751 | 0,058 |

| 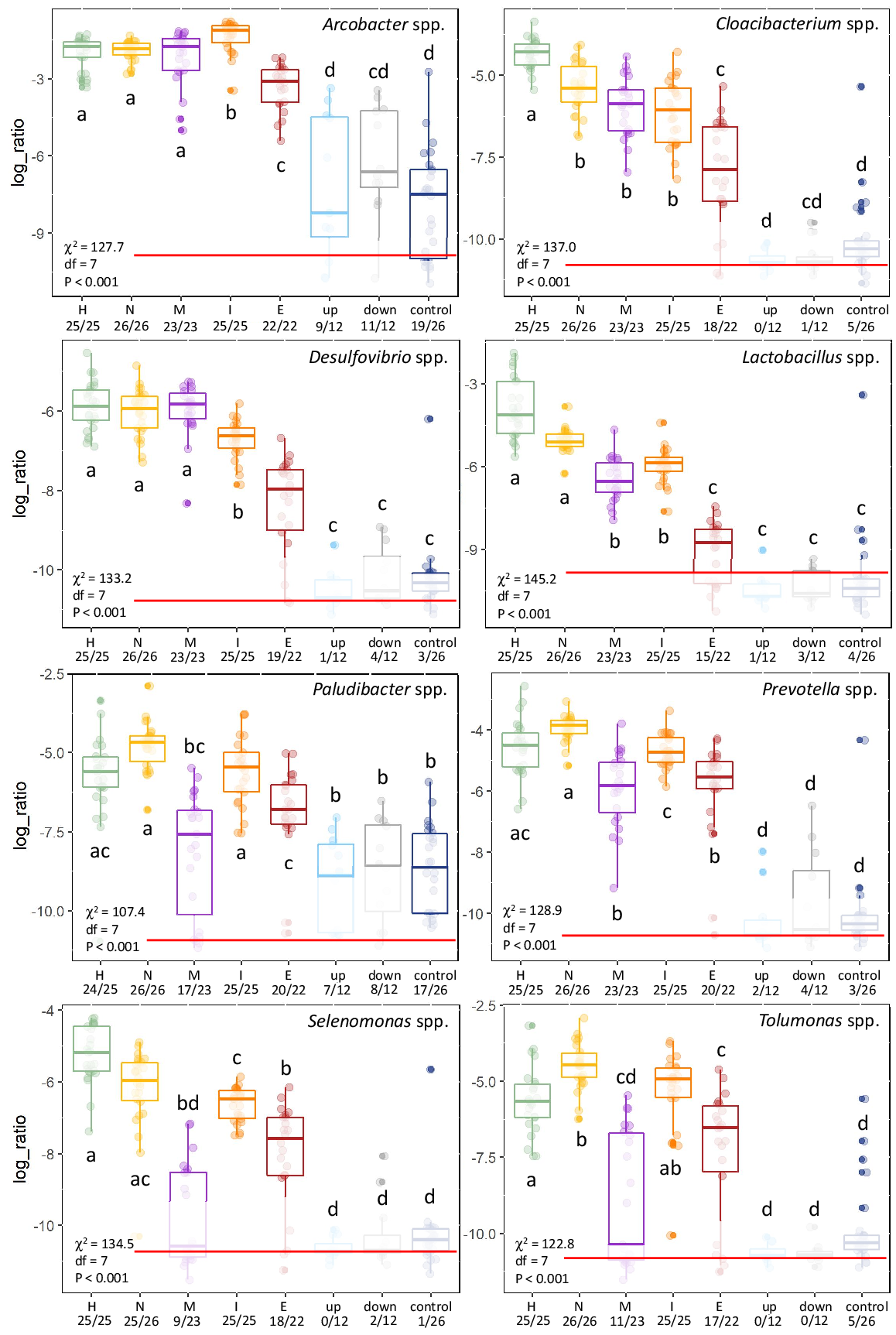 |
| --- |
| **Figure S4. Pathway of genera including clinical enriched genera.** Relative abundance of genera along the wastewater pathway. The Kruskal-Wallis statistics are shown on the left side of each panel, for all species there was significant difference in abundance observed between two or more locations. Group differences were assessed Dunn’s test with P value adjustment method: BH. The red line represents the average detection limit. Samples below the detection limit are faded. H = hospital, N = nursing home, M = municipal, I = influent, E = effluent, up = upstream surface water, down = downstream surface water, and control = control surface water. |

| 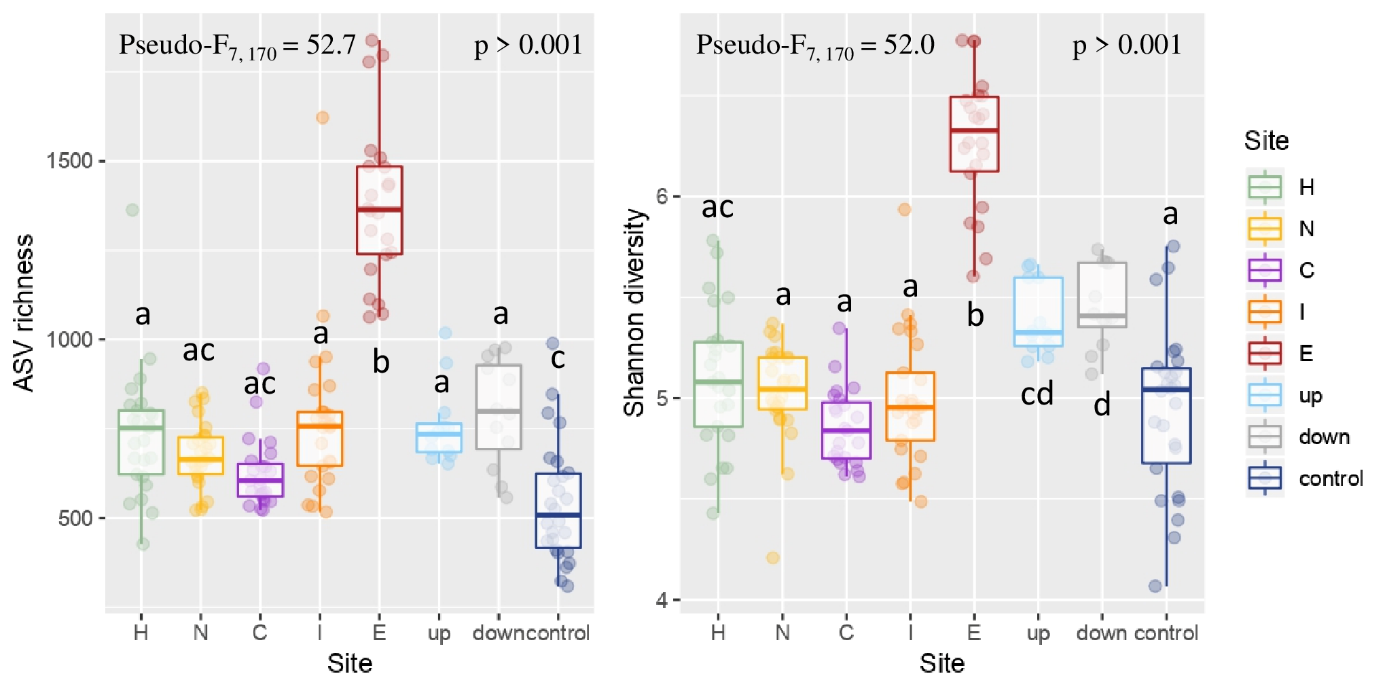 |
| --- |
| **Figure S5. ASV richness and Shannon diversity across all sites.** Both ASV richness (left) and Shannon diversity (right) show that effluent was the location with the highest diversity. Group differences were assessed by Tukey HSD. H = hospital, N = nursing home, C = community (municipal), I = influent, E = effluent, Up = upstream surface water, Down = downstream surface water, and Control = control surface water. |
