# Supplementary material for: Effects of Clinical Wastewater on the Bacterial Community Structure from Sewage to the Environment
Source: Microorganisms. 2021 Mar 31;9(4):718. doi: 10.3390/microorganisms9040718 (PMC8065902; doi:10.3390/microorganisms9040718)
Supplement: Supplementary file 1 [file microorganisms-09-00718-s001.zip › Supplementary material.pdf]

Supplementary material

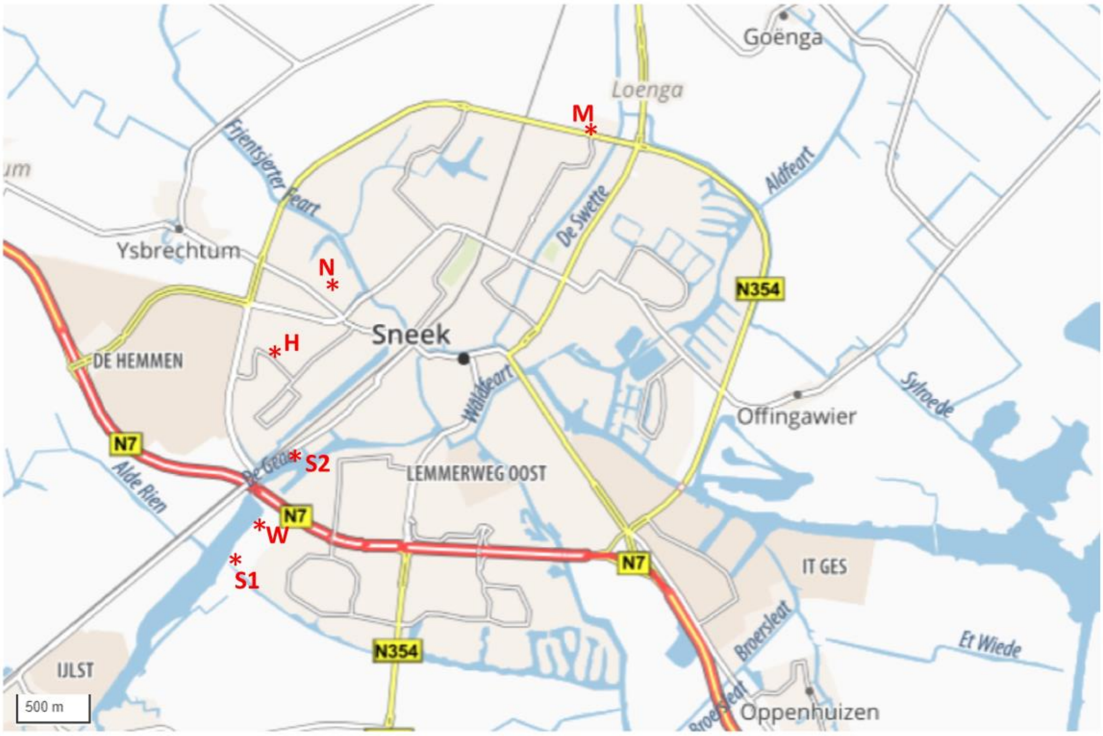

Figure S1. Locations of the sampling points in Sneek. H = hospital, N = nursing home, C = community, W = WWTP (influent and effluent), S1 and S2 = Receiving surface water. The location of the control surface water sample is not indicated. The map of Sneek is obtained from viamichelin.nl.

Table S1a. Statistical analysis of weighted UniFrac distances along the wastewater pathway. The p-values of the PERMANOVA test are shown below the diagonal (Adjustment method for p-value: FDR), and the permuted p-values using permutation test for homogeneity of multivariate dispersion are shown above the diagonal.

	**Hospital**	**Nursing-home**	**Community**	**Influent**	**Effluent**
**Hospital**	X	0.800	0.233	0.218	0.775
**Nursing-home**	**0.001**	X	0.201	0.439	0.605
**Community**	**0.001**	**0.001**	X	**0.010**	0.461
**Influent**	**0.001**	**0.001**	**0.001**	X	0.115
**Effluent**	**0.001**	**0.001**	**0.001**	**0.001**	X

Table S1b. Statistical analysis of unweighted UniFrac distances along the wastewater pathway. The p-values of the PERMANOVA test are shown below the diagonal (Adjustment method for p-value: FDR), and the permuted p-values using permutation test for homogeneity of multivariate dispersion are shown above the diagonal.

	**Hospital**	**Nursing-home**	**Community**	**Influent**	**Effluent**
**Hospital**	X	**0.014**	**0,009**	**0.018**	**0.001**
**Nursing-home**	**0.001**	X	0.554	0.684	**0.001**
**Community**	**0.001**	**0.001**	X	0.970	**0.001**
**Influent**	**0.001**	**0.001**	**0.001**	X	**0.001**
**Effluent**	**0.001**	**0.001**	**0.001**	**0.001**	X

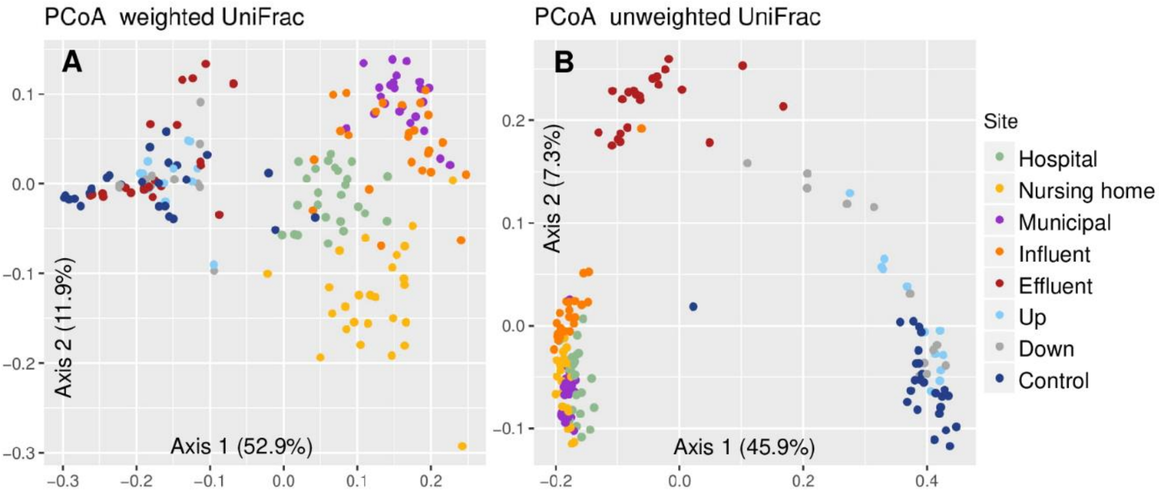

Figure S2. Bacterial beta diversity off all the locations. Principal coordinates ordination of different wastewaters based on weighted UniFrac distances (A) and unweighted UniFrac distances (B). 69% of the diversity in bacterial composition and 59% of the diversity in bacterial membership could be explained by the locations. The percentage of variation explained by each axis is shown between parentheses. H = hospital, N = nursing home, C = community, I = influent, E = effluent, Up = upstream surface water, Down = downstream surface water, and Control = control surface water.

Table S2a. Statistical analysis of weighted UniFrac distances along all the locations. The p-values of the PERMANOVA test are shown below the diagonal (Adjustment method for p-value: FDR), and the permuted p-values using permutation test for homogeneity of multivariate dispersion are shown above the diagonal.

	**Hospital**	**Nursing-home**	**Community**	**Influent**	**Effluent**	**Up-stream**	**Down-stream**	**Control**
**Hospital**	X	0.784	0.219	0.202	0.565	0.742	0.886	**0.024**
**Nursing-home**	**0.001**	X	0.215	0.419	0.448	0.605	0.927	0.090
**Community**	**0.001**	**0.001**	X	**0.007**	0.771	0.525	0.275	**0.004**
**Influent**	**0.001**	**0.001**	**0.001**	X	0.121	0.141	0.397	0.162
**Effluent**	**0.001**	**0.001**	**0.001**	**0.001**	X	0.896	0.613	**0.023**
**Up-stream**	**0.001**	**0.001**	**0.001**	**0.001**	**0.001**	X	0.717	0.077
**Down-stream**	**0.001**	**0.001**	**0.001**	**0.001**	**0.001**	0.967	X	0.132
**Control**	**0.001**	**0.001**	**0.001**	**0.001**	**0.001**	**0.042**	0.085	X

Table S2b. Statistical analysis of unweighted UniFrac distances along all the locations. The p-values of the PERMANOVA test are shown below the diagonal (Adjustment method for p-value: FDR), and the permuted p-values using permutation test for homogeneity of multivariate dispersion are shown above the diagonal.

	**Hospital**	**Nursing-home**	**Community**	**Influent**	**Effluent**	**Up-stream**	**Down-stream**	**Control**
**Hospital**	X	**0.024**	**0.002**	**0.019**	**0.001**	**0.001**	**0.001**	**0.001**
**Nursing-home**	**0.001**	X	0.542	0.678	**0.001**	**0.001**	**0.001**	**0.001**
**Community**	**0.001**	**0.001**	X	0.962	**0.001**	**0.001**	**0.001**	**0.001**
**Influent**	**0.001**	**0.001**	**0.001**	X	**0.001**	**0.001**	**0.001**	**0.001**
**Effluent**	**0.001**	**0.001**	**0.001**	**0.001**	X	**0.002**	**0.001**	**0.001**
**Up-stream**	**0.001**	**0.001**	**0.001**	**0.001**	**0.001**	X	0.329	0.819
**Down-stream**	**0.001**	**0.001**	**0.001**	**0.001**	**0.001**	0.754	X	0.467
**Control**	**0.001**	**0.001**	**0.001**	**0.001**	**0.001**	**0.001**	**0.001**	X

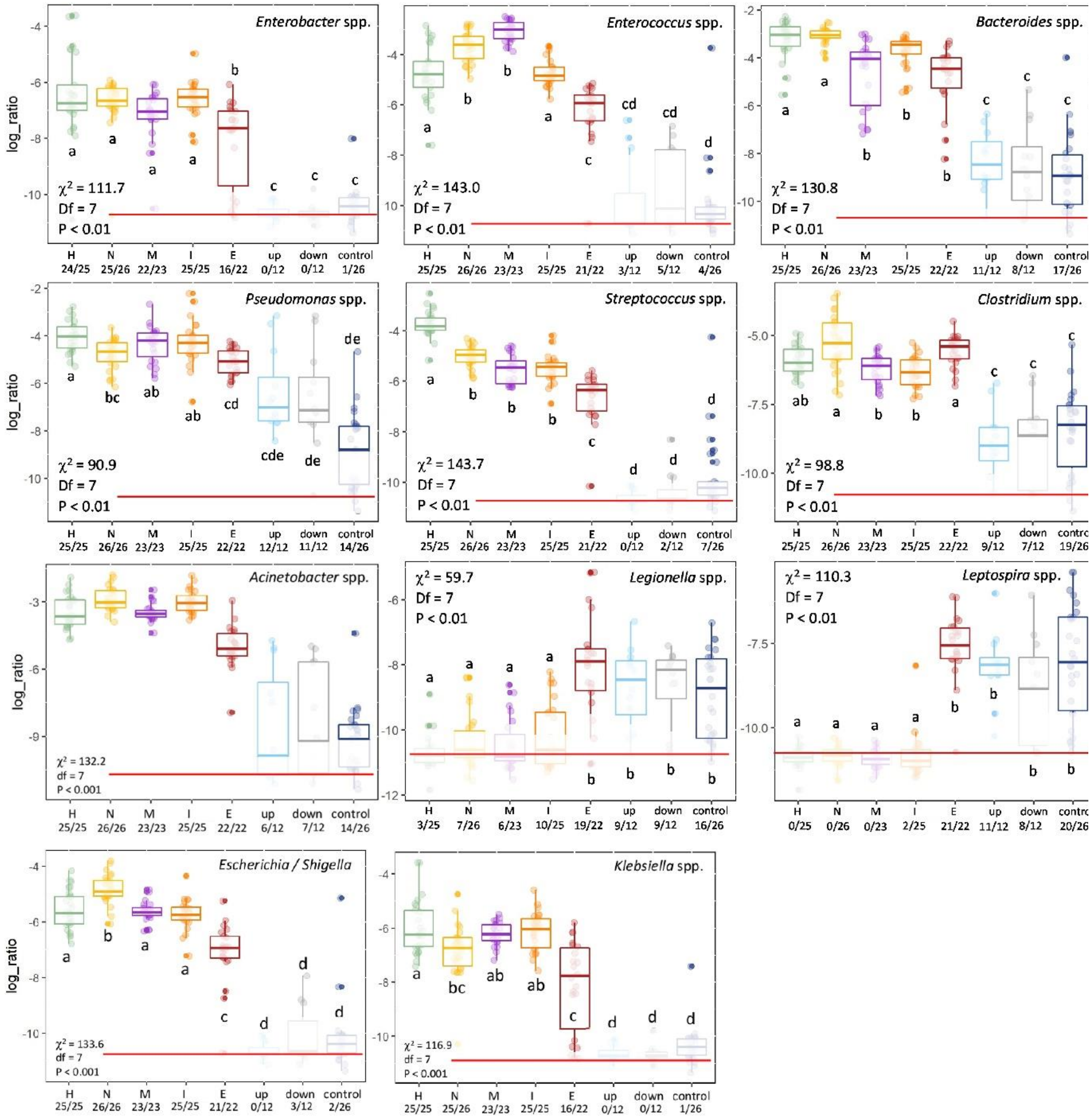

Figure S3. Target genera based on pathogenic potential. Relative abundance of target genera along the wastewater pathway. The Kruskal-Wallis statistics are shown on the left side of each panel, for all species there was significant difference in abundance observed between two or more locations. Group differences were assessed Dunn's test with P value adjustment method: BH. The red line represents the average detection limit. Samples below the detection limit are faded. H = hospital, N = nursing home, M = municipal, I = influent, E = effluent, up = upstream surface water, down = downstream surface water, and control = control surface water.

Table S3. Clinical enriched bacteria. 207 taxa were identified to be significantly more abundant in both hospital (H) and nursing-home (N) wastewater when compared to community (C) wastewater. Taxa belonging to the target genera that include potential pathogenic genera are shown in red.

Bacterial identification					Mean abundance (%)			Log2-FoldChange		p-value	
**Class**	**Order**	**Family**	**Genus**	**Species**	**H**	**N**	**C**	**H - C**	**N - C**	**H - C**	**N - C**
Actinobacteria	Actinomycetales	Actinomycetaceae	Actinomyces	NA	0,041	0,069	0,001	3,402	3,983	0,000	0,000
Actinobacteria	Micrococcales	Bogoriellaceae	Georgenia	NA	0,012	0,016	0,001	3,071	3,595	0,000	0,000
Actinobacteria	Micrococcales	Micrococcaceae	Glutamicibacter	NA	0,018	0,013	0,003	2,637	2,239	0,020	0,058
Actinobacteria	NA	NA	NA	NA	0,005	0,028	0,000	2,190	4,115	0,020	0,000
Actinobacteria	Propionibacteriales	Propionibacteriaceae	Propioniciclava	NA	0,023	0,010	0,000	3,581	2,551	0,005	0,063
Coriobacteriia	Coriobacteriales	Coriobacteriaceae	Enterorhabdus	NA	0,027	0,027	0,028	0,635	0,702	0,075	0,043
Coriobacteriia	Coriobacteriales	Coriobacteriaceae	Gordonibacter	NA	0,020	0,032	0,003	2,737	3,133	0,000	0,000
Coriobacteriia	Coriobacteriales	Coriobacteriaceae	NA	NA	0,004	0,009	0,001	2,066	2,858	0,037	0,001
Bacteroidia	Bacteroidales	Bacteroidaceae	Bacteroides	clarus	0,017	0,044	0,000	3,086	4,277	0,000	0,000
Bacteroidia	Bacteroidales	Bacteroidaceae	Bacteroides	coprocola	0,067	0,072	0,013	1,890	1,761	0,000	0,001
Bacteroidia	Bacteroidales	Bacteroidaceae	Bacteroides	eggerthii	0,062	0,064	0,007	2,193	2,443	0,000	0,000
Bacteroidia	Bacteroidales	Bacteroidaceae	Bacteroides	NA	0,046	0,092	0,001	3,964	4,522	0,000	0,000
Bacteroidia	Bacteroidales	Bacteroidaceae	Bacteroides	NA	0,232	0,187	0,015	3,292	3,031	0,000	0,000
Bacteroidia	Bacteroidales	Bacteroidaceae	Bacteroides	NA	0,044	0,273	0,003	3,050	3,883	0,000	0,000
Bacteroidia	Bacteroidales	Bacteroidaceae	Bacteroides	NA	0,068	0,060	0,012	2,140	2,063	0,001	0,002
Bacteroidia	Bacteroidales	Bacteroidaceae	Bacteroides	NA	0,074	0,072	0,017	1,639	1,819	0,000	0,000
Bacteroidia	Bacteroidales	Bacteroidaceae	Bacteroides	NA	0,117	0,122	0,031	1,225	1,313	0,000	0,000
Bacteroidia	Bacteroidales	Bacteroidaceae	Bacteroides	NA	0,350	0,371	0,096	0,943	1,080	0,000	0,000
Bacteroidia	Bacteroidales	Bacteroidaceae	Bacteroides	stercoris	0,114	0,067	0,002	4,068	4,095	0,000	0,000
Bacteroidia	Bacteroidales	Bacteroidaceae	Bacteroides	thetaiotaomicron	0,091	0,077	0,008	2,399	2,469	0,000	0,000
Bacteroidia	Bacteroidales	Bacteroidaceae	Bacteroides	uniformis	0,174	0,316	0,063	1,158	1,382	0,000	0,000
Bacteroidia	Bacteroidales	Bacteroidaceae	Bacteroides	uniformis	0,271	0,408	0,053	1,143	1,384	0,000	0,000
Bacteroidia	Bacteroidales	Bacteroidaceae	Bacteroides	vulgatus	0,245	0,377	0,079	1,104	1,352	0,000	0,000
Bacteroidia	Bacteroidales	Bacteroidaceae	Bacteroides	vulgatus	0,378	0,461	0,132	0,731	0,800	0,000	0,000
Bacteroidia	Bacteroidales	Bacteroidales_S24-7_group	NA	NA	0,035	0,028	0,001	3,494	3,318	0,000	0,000
Bacteroidia	Bacteroidales	Bacteroidales_S24-7_group	NA	NA	0,008	0,073	0,001	2,387	4,226	0,000	0,000
Bacteroidia	Bacteroidales	M2PB4-65_termite_group	NA	NA	0,019	0,025	0,000	3,015	4,025	0,001	0,000
Bacteroidia	Bacteroidales	Porphyromonadaceae	Barnesiella	NA	0,030	0,058	0,007	1,777	2,452	0,058	0,005
Bacteroidia	Bacteroidales	Porphyromonadaceae	Coprobacter	NA	0,016	0,023	0,001	3,125	3,738	0,000	0,000
Bacteroidia	Bacteroidales	Porphyromonadaceae	Dysgonomonas	oryzarvi	0,060	0,044	0,012	2,477	2,155	0,000	0,000
Bacteroidia	Bacteroidales	Porphyromonadaceae	Macellibacteroides	fermentans	0,270	0,309	0,218	0,386	0,386	0,070	0,070
Bacteroidia	Bacteroidales	Porphyromonadaceae	Macellibacteroides	NA	0,012	0,074	0,002	2,250	4,121	0,004	0,000

Bacteroidia	Bacteroidales	Porphyromonadaceae	Microbacter	NA	0,031	0,058	0,001	3,654	4,233	0,000	0,000
Bacteroidia	Bacteroidales	Porphyromonadaceae	Microbacter	NA	0,009	0,044	0,000	3,259	4,248	0,000	0,000
Bacteroidia	Bacteroidales	Porphyromonadaceae	NA	NA	0,011	0,015	0,000	3,126	3,494	0,000	0,000
Bacteroidia	Bacteroidales	Porphyromonadaceae	NA	NA	0,058	0,052	0,018	1,086	1,254	0,014	0,003
Bacteroidia	Bacteroidales	Porphyromonadaceae	Odoribacter	splanchnicus	0,032	0,056	0,006	2,014	2,556	0,000	0,000
Bacteroidia	Bacteroidales	Porphyromonadaceae	Odoribacter	splanchnicus	0,044	0,058	0,007	1,796	2,299	0,001	0,000
Bacteroidia	Bacteroidales	Porphyromonadaceae	Paludibacter	NA	0,023	0,020	0,000	3,526	3,760	0,000	0,000
Bacteroidia	Bacteroidales	Porphyromonadaceae	Paludibacter	NA	0,104	0,193	0,008	2,689	3,051	0,000	0,000
Bacteroidia	Bacteroidales	Porphyromonadaceae	Paludibacter	NA	0,010	0,158	0,002	1,696	4,253	0,054	0,000
Bacteroidia	Bacteroidales	Porphyromonadaceae	Paludibacter	NA	0,166	0,067	0,034	1,276	0,809	0,000	0,025
Bacteroidia	Bacteroidales	Porphyromonadaceae	Paludibacter	NA	0,164	0,345	0,024	1,223	1,737	0,000	0,000
Bacteroidia	Bacteroidales	Porphyromonadaceae	Parabacteroides	distasonis	0,081	0,109	0,022	1,943	2,098	0,000	0,000
Bacteroidia	Bacteroidales	Porphyromonadaceae	Parabacteroides	distasonis	0,021	0,034	0,007	1,801	2,061	0,002	0,000
Bacteroidia	Bacteroidales	Porphyromonadaceae	Parabacteroides	goldsteinii	0,011	0,060	0,000	2,797	4,477	0,000	0,000
Bacteroidia	Bacteroidales	Porphyromonadaceae	Parabacteroides	merdae	0,161	0,224	0,114	0,427	0,560	0,064	0,009
Bacteroidia	Bacteroidales	Porphyromonadaceae	Parabacteroides	NA	0,080	0,011	0,002	3,942	2,156	0,000	0,004
Bacteroidia	Bacteroidales	Porphyromonadaceae	Proteiniphilum	NA	0,003	0,016	0,000	1,889	3,716	0,059	0,000
Bacteroidia	Bacteroidales	Prevotellaceae	Paraprevotella	NA	0,024	0,050	0,001	3,231	4,130	0,000	0,000
Bacteroidia	Bacteroidales	Prevotellaceae	Paraprevotella	NA	0,033	0,019	0,004	2,813	2,465	0,000	0,000
Bacteroidia	Bacteroidales	Prevotellaceae	Prevotella_9	NA	1,061	0,938	0,011	4,128	4,223	0,000	0,000
Bacteroidia	Bacteroidales	Prevotellaceae	Prevotella_9	NA	0,023	0,029	0,000	2,969	3,577	0,028	0,005
Bacteroidia	Bacteroidales	Prevotellaceae	Prevotella_9	NA	0,076	0,104	0,018	1,848	2,003	0,013	0,006
Bacteroidia	Bacteroidales	Prevotellaceae	Prevotella_9	NA	0,008	0,130	0,002	1,755	4,406	0,026	0,000
Bacteroidia	Bacteroidales	Prevotellaceae	Prevotellaceae_NK3B31_group	NA	0,029	0,019	0,001	3,479	2,740	0,001	0,015
Bacteroidia	Bacteroidales	Prolixibacteraceae	Prolixibacter	NA	0,021	0,014	0,000	3,611	3,291	0,000	0,000
Bacteroidia	Bacteroidales	Prolixibacteraceae	Prolixibacter	NA	0,010	0,009	0,000	2,554	2,410	0,045	0,062
Bacteroidia	Bacteroidales	Rikenellaceae	Alistipes	finegoldii	0,031	0,048	0,000	3,971	4,480	0,000	0,000
Bacteroidia	Bacteroidales	Rikenellaceae	Alistipes	inops	0,030	0,034	0,007	1,815	2,075	0,000	0,000
Bacteroidia	Bacteroidales	Rikenellaceae	Alistipes	NA	0,046	0,018	0,002	3,258	2,322	0,000	0,003
Bacteroidia	Bacteroidales	Rikenellaceae	Alistipes	NA	0,005	0,005	0,000	2,445	2,546	0,096	0,082
Bacteroidia	Bacteroidales	Rikenellaceae	Alistipes	NA	0,015	0,014	0,003	2,414	2,317	0,024	0,032
Bacteroidia	Bacteroidales	Rikenellaceae	Alistipes	NA	0,129	0,169	0,015	1,861	2,109	0,000	0,000
Bacteroidia	Bacteroidales	Rikenellaceae	Alistipes	NA	0,014	0,011	0,004	1,791	1,893	0,023	0,014
Bacteroidia	Bacteroidales	Rikenellaceae	Alistipes	obesi	0,024	0,024	0,004	2,062	2,372	0,000	0,000
Bacteroidia	Bacteroidales	Rikenellaceae	Alistipes	putredinis	0,258	0,251	0,040	1,071	1,131	0,000	0,000
Bacteroidia	Bacteroidales	Rikenellaceae	Alistipes	shahii	0,035	0,043	0,000	3,757	4,095	0,000	0,000
Bacteroidia	Bacteroidales	Rikenellaceae	Anaerocella	delicata	0,101	0,057	0,001	4,270	4,090	0,000	0,000
Bacteroidia	Bacteroidales	Rikenellaceae	dgA-11_gut_group	NA	0,392	0,017	0,008	2,790	1,366	0,000	0,001
Bacteroidia	Bacteroidales	Rikenellaceae	vadinBC27_wastewater-sludge_group	NA	0,057	0,039	0,007	2,162	2,013	0,000	0,000
Bacteroidia	Bacteroidales	Rikenellaceae	vadinBC27_wastewater-sludge_group	NA	0,016	0,024	0,007	1,281	1,772	0,022	0,001
Bacteroidia	Bacteroidales	Rikenellaceae	vadinBC27_wastewater-sludge_group	NA	0,099	0,039	0,025	0,835	0,608	0,004	0,055

Flavobacteriia	Flavobacteriales	Flavobacteriaceae	Cloacibacterium	normanense	1,412	0,542	0,339	0,625	0,337	0,000	0,098
Flavobacteriia	Flavobacteriales	Flavobacteriaceae	Flavobacterium	NA	0,015	0,041	0,002	2,755	3,668	0,000	0,000
Flavobacteriia	Flavobacteriales	Flavobacteriaceae	Flavobacterium	NA	0,141	0,092	0,020	1,817	1,688	0,000	0,000
Sphingobacteriia	Sphingobacteriales	KD1-131	NA	NA	0,004	0,018	0,000	1,888	3,825	0,045	0,000
Sphingobacteriia	Sphingobacteriales	ST-12K33	NA	NA	0,041	0,017	0,004	2,753	2,102	0,005	0,045
Sphingobacteriia	Sphingobacteriales	ST-12K33	NA	NA	0,096	0,033	0,036	1,479	0,722	0,000	0,068
Chlorobia	Chlorobiales	OPB56	NA	NA	0,022	0,015	0,001	2,792	3,194	0,001	0,000
Melainabacteria	Gastranaerophilales	NA	NA	NA	0,008	0,022	0,002	1,580	2,779	0,073	0,000
Fibrobacteria	Fibrobacterales	Fibrobacteraceae	possible_genus_06	NA	0,055	0,030	0,001	4,098	3,612	0,000	0,000
Fibrobacteria	Fibrobacterales	Fibrobacteraceae	possible_genus_06	NA	0,044	0,006	0,002	3,651	2,020	0,000	0,030
Bacilli	Lactobacillales	Lactobacillaceae	Lactobacillus	delbrueckii	0,889	0,041	0,002	4,867	2,540	0,000	0,000
Bacilli	Lactobacillales	Lactobacillaceae	Lactobacillus	delbrueckii	1,278	0,301	0,013	4,189	3,898	0,000	0,000
Bacilli	Lactobacillales	Lactobacillaceae	Lactobacillus	NA	0,096	0,040	0,001	3,822	4,070	0,000	0,000
Bacilli	Lactobacillales	Lactobacillaceae	Lactobacillus	NA	0,005	0,029	0,001	1,565	3,652	0,051	0,000
Bacilli	Lactobacillales	Streptococcaceae	Lactococcus	NA	0,492	3,768	0,074	0,921	1,442	0,000	0,000
Bacilli	Lactobacillales	Streptococcaceae	Lactococcus	NA	0,269	0,970	0,039	0,920	1,443	0,000	0,000
Bacilli	Lactobacillales	Streptococcaceae	Lactococcus	raffinolactis	0,321	1,994	0,079	1,448	2,043	0,000	0,000
Bacilli	Lactobacillales	Streptococcaceae	Streptococcus	NA	1,384	0,219	0,067	1,147	0,448	0,000	0,062
Clostridia	Clostridiales	Christensenellaceae	Christensenellaceae_R-7_group	NA	0,068	0,010	0,000	4,445	2,646	0,000	0,009
Clostridia	Clostridiales	Christensenellaceae	Christensenellaceae_R-7_group	NA	0,010	0,019	0,002	2,693	3,253	0,000	0,000
Clostridia	Clostridiales	Christensenellaceae	Christensenellaceae_R-7_group	NA	0,041	0,079	0,005	2,327	2,810	0,000	0,000
Clostridia	Clostridiales	Christensenellaceae	Christensenellaceae_R-7_group	NA	0,089	0,047	0,021	1,293	1,112	0,000	0,000
Clostridia	Clostridiales	Christensenellaceae	Christensenellaceae_R-7_group	NA	0,021	0,047	0,015	0,612	1,220	0,084	0,000
Clostridia	Clostridiales	Clostridiaceae_1	Proteiniclasticum	ruminis	0,083	0,096	0,005	3,239	3,354	0,000	0,000
Clostridia	Clostridiales	Eubacteriaceae	Anaerofustis	NA	0,010	0,007	0,002	2,379	1,944	0,002	0,017
Clostridia	Clostridiales	Eubacteriaceae	NA	NA	3,518	0,147	0,049	1,778	0,840	0,000	0,000
Clostridia	Clostridiales	Family_XI	NA	NA	0,020	0,040	0,010	1,159	1,914	0,010	0,000
Clostridia	Clostridiales	Family_XI	Tissierella	NA	0,025	0,009	0,000	3,992	3,131	0,000	0,000
Clostridia	Clostridiales	Family_XI	Tissierella	NA	0,013	0,045	0,001	2,442	3,754	0,000	0,000
Clostridia	Clostridiales	Lachnospiraceae	Butyrivibrio	crossotus	0,027	0,050	0,005	2,227	2,673	0,000	0,000
Clostridia	Clostridiales	Lachnospiraceae	Coprococcus_2	eutactus	0,057	0,103	0,005	2,402	3,113	0,000	0,000
Clostridia	Clostridiales	Lachnospiraceae	Coprococcus_2	NA	0,041	0,010	0,001	3,899	2,524	0,000	0,000
Clostridia	Clostridiales	Lachnospiraceae	Fusicatenibacter	NA	0,034	0,046	0,013	1,333	1,603	0,000	0,000
Clostridia	Clostridiales	Lachnospiraceae	Howardella	NA	0,003	0,010	0,002	1,545	2,994	0,080	0,000
Clostridia	Clostridiales	Lachnospiraceae	NA	NA	0,098	0,049	0,000	4,898	4,200	0,000	0,000
Clostridia	Clostridiales	Lachnospiraceae	NA	NA	0,029	0,046	0,003	2,641	2,965	0,000	0,000
Clostridia	Clostridiales	Lachnospiraceae	NA	NA	0,008	0,007	0,002	2,295	2,076	0,016	0,032
Clostridia	Clostridiales	Lachnospiraceae	NA	NA	0,047	0,101	0,015	1,246	1,736	0,000	0,000
Clostridia	Clostridiales	Lachnospiraceae	NA	NA	0,018	0,027	0,009	1,039	1,475	0,093	0,009
Clostridia	Clostridiales	Lachnospiraceae	NA	NA	0,196	0,132	0,055	0,911	0,729	0,000	0,006
Clostridia	Clostridiales	Lachnospiraceae	Roseburia	intestinalis	0,031	0,024	0,004	2,928	2,630	0,016	0,033
Clostridia	Clostridiales	Lachnospiraceae	Roseburia	NA	0,020	0,106	0,011	0,909	2,152	0,079	0,000
Clostridia	Clostridiales	Ruminococcaceae	Ercella	NA	0,023	0,030	0,004	2,589	2,803	0,000	0,000

Clostridia	Clostridiales	Ruminococcaceae	Intestinimonas	NA	0,009	0,012	0,000	2,927	3,134	0,022	0,012
Clostridia	Clostridiales	Ruminococcaceae	Intestinimonas	NA	0,011	0,019	0,000	2,713	3,848	0,006	0,000
Clostridia	Clostridiales	Ruminococcaceae	Intestinimonas	NA	0,007	0,018	0,001	2,059	3,405	0,023	0,000
Clostridia	Clostridiales	Ruminococcaceae	NA	NA	0,015	0,057	0,000	3,659	4,585	0,000	0,000
Clostridia	Clostridiales	Ruminococcaceae	NA	NA	0,010	0,020	0,000	2,720	3,783	0,001	0,000
Clostridia	Clostridiales	Ruminococcaceae	NA	NA	0,005	0,012	0,000	2,259	3,324	0,070	0,003
Clostridia	Clostridiales	Ruminococcaceae	NA	NA	0,023	0,035	0,010	1,111	1,662	0,009	0,000
Clostridia	Clostridiales	Ruminococcaceae	NA	NA	0,024	0,020	0,010	0,987	0,965	0,057	0,064
Clostridia	Clostridiales	Ruminococcaceae	NA	NA	0,067	0,112	0,039	0,578	0,793	0,025	0,001
Clostridia	Clostridiales	Ruminococcaceae	Ruminiclostridium_5	NA	0,016	0,048	0,004	1,937	2,978	0,000	0,000
Clostridia	Clostridiales	Ruminococcaceae	Ruminiclostridium_6	NA	0,043	0,102	0,001	4,025	4,572	0,000	0,000
Clostridia	Clostridiales	Ruminococcaceae	Ruminococcaceae_NK4A214_group	NA	0,020	0,014	0,004	2,291	1,850	0,001	0,011
Clostridia	Clostridiales	Ruminococcaceae	Ruminococcaceae_UCG-002	NA	0,006	0,033	0,000	1,969	4,062	0,093	0,000
Clostridia	Clostridiales	Ruminococcaceae	Ruminococcaceae_UCG-002	NA	0,097	0,112	0,047	0,584	0,731	0,021	0,002
Clostridia	Clostridiales	Ruminococcaceae	Ruminococcaceae_UCG-002	NA	0,117	0,160	0,103	0,405	0,543	0,096	0,016
Clostridia	Clostridiales	Ruminococcaceae	Ruminococcaceae_UCG-004	NA	0,008	0,020	0,004	1,294	2,608	0,060	0,000
Clostridia	Clostridiales	Ruminococcaceae	Ruminococcaceae_UCG-005	NA	0,013	0,018	0,000	2,764	3,447	0,010	0,001
Clostridia	Clostridiales	Ruminococcaceae	Ruminococcaceae_UCG-010	NA	0,005	0,006	0,002	2,084	2,360	0,015	0,004
Clostridia	Clostridiales	Ruminococcaceae	Ruminococcaceae_UCG-014	NA	0,035	0,117	0,008	2,031	2,554	0,000	0,000
Clostridia	Clostridiales	Ruminococcaceae	Ruminococcaceae_UCG-014	NA	0,046	0,047	0,026	0,689	0,562	0,019	0,069
Clostridia	Clostridiales	Ruminococcaceae	Ruminococcus_1	bicirculans	0,086	0,073	0,002	3,944	3,830	0,000	0,000
Clostridia	Clostridiales	Ruminococcaceae	Ruminococcus_1	NA	0,009	0,012	0,000	2,970	3,202	0,010	0,004
Clostridia	Clostridiales	Ruminococcaceae	Ruminococcus_1	NA	0,004	0,018	0,000	2,045	3,988	0,017	0,000
Clostridia	Clostridiales	Ruminococcaceae	Ruminococcus_2	bromii	0,084	0,150	0,023	1,259	1,777	0,000	0,000
Clostridia	Clostridiales	Ruminococcaceae	Ruminococcus_2	bromii	0,183	0,126	0,081	0,806	0,570	0,000	0,020
Clostridia	Clostridiales	Ruminococcaceae	Ruminococcus_2	NA	0,110	0,071	0,031	1,124	1,038	0,001	0,002
Erysipelotrichia	Erysipelotrichales	Erysipelotrichaceae	Catenisphaera	NA	0,018	0,024	0,007	1,525	2,066	0,023	0,001
Erysipelotrichia	Erysipelotrichales	Erysipelotrichaceae	Erysipelothrix	NA	0,045	0,018	0,004	3,035	1,753	0,000	0,032
Erysipelotrichia	Erysipelotrichales	Erysipelotrichaceae	Erysipelothrix	NA	0,008	0,009	0,001	2,553	2,756	0,002	0,001
Erysipelotrichia	Erysipelotrichales	Erysipelotrichaceae	Faecalitalea	cylindroides	0,005	0,015	0,003	1,676	2,694	0,045	0,000
Erysipelotrichia	Erysipelotrichales	Erysipelotrichaceae	Faecalitalea	NA	0,005	0,009	0,000	2,326	2,984	0,097	0,023
Erysipelotrichia	Erysipelotrichales	Erysipelotrichaceae	NA	NA	0,009	0,013	0,001	2,548	2,847	0,037	0,016
Negativicutes	Selenomonadales	Acidaminococcaceae	Phascolarctobacterium	NA	0,028	0,019	0,007	2,123	1,620	0,001	0,014
Negativicutes	Selenomonadales	Veillonellaceae	Anaeroarcus	burkinensis	0,083	0,071	0,004	3,073	2,925	0,000	0,000
Negativicutes	Selenomonadales	Veillonellaceae	Anaeroarcus	NA	0,237	0,204	0,036	1,090	0,995	0,000	0,000
Negativicutes	Selenomonadales	Veillonellaceae	Megasphaera	NA	0,126	0,040	0,001	4,217	3,448	0,000	0,000
Negativicutes	Selenomonadales	Veillonellaceae	NA	NA	0,043	0,046	0,023	0,868	1,039	0,005	0,000
Negativicutes	Selenomonadales	Veillonellaceae	Selenomonas	lacticifex	0,070	0,120	0,006	2,733	2,996	0,000	0,000
Negativicutes	Selenomonadales	Veillonellaceae	Veillonella	NA	0,250	0,287	0,001	4,295	4,773	0,000	0,000
Fusobacteriia	Fusobacteriales	Leptotrichiaceae	NA	NA	0,303	0,047	0,004	4,138	3,259	0,000	0,000
Bacteria beloning tot he phylum Gracilibacteria					0,078	0,037	0,008	2,052	1,806	0,000	0,000
Lentisphaeria	Victivallales	Victivallaceae	Victivallis	NA	0,010	0,012	0,004	1,337	1,797	0,095	0,015
Alphaproteobacteria	Rhodospirillales	Rhodospirillaceae	Elstera	NA	0,018	0,007	0,002	3,026	2,273	0,000	0,009

Alphaproteobacteria	Rhodospirillales	Rhodospirillaceae	NA	NA	0,014	0,014	0,004	1,621	1,237	0,017	0,088
Alphaproteobacteria	Rhodospirillales	Rhodospirillaceae	NA	NA	0,045	0,045	0,020	1,023	1,129	0,003	0,001
Betaproteobacteria	Burkholderiales	Alcaligenaceae	Sutterella	NA	0,006	0,046	0,000	2,555	4,339	0,004	0,000
Betaproteobacteria	Burkholderiales	Comamonadaceae	Acidovorax	NA	0,032	0,044	0,007	2,462	2,693	0,001	0,000
Betaproteobacteria	Burkholderiales	Comamonadaceae	Diaphorobacter	NA	0,163	0,075	0,039	1,540	1,125	0,000	0,000
Betaproteobacteria	DR-16	NA	NA	NA	0,684	0,093	0,000	5,237	4,495	0,000	0,000
Betaproteobacteria	Neisseriales	Neisseriaceae	Formivibrio	NA	0,026	0,149	0,003	2,521	3,433	0,000	0,000
Betaproteobacteria	Neisseriales	Neisseriaceae	Laribacter	hongkongensis	0,081	0,035	0,019	2,057	1,437	0,000	0,000
Betaproteobacteria	Neisseriales	Neisseriaceae	Microvirgula	aerodenitrificans	0,012	0,040	0,001	3,069	3,871	0,000	0,000
Betaproteobacteria	Rhodocyclales	Rhodocyclaceae	Dechlorobacter	NA	0,262	0,212	0,106	1,164	0,986	0,000	0,000
Betaproteobacteria	Rhodocyclales	Rhodocyclaceae	Dechloromonas	NA	0,511	0,168	0,032	1,671	1,388	0,000	0,000
Betaproteobacteria	Rhodocyclales	Rhodocyclaceae	Propionivibrio	NA	0,061	0,017	0,000	4,357	3,322	0,000	0,000
Betaproteobacteria	Rhodocyclales	Rhodocyclaceae	Propionivibrio	NA	0,345	0,090	0,053	1,513	0,956	0,000	0,000
Deltaproteobacteria	Desulfobacterales	Desulfobulbaceae	Desulfobulbus	NA	0,032	0,006	0,000	3,995	2,380	0,000	0,033
Deltaproteobacteria	Desulfovibrionales	Desulfovibrionaceae	Desulfovibrio	desulfuricans	0,205	0,099	0,086	0,728	0,426	0,001	0,094
Deltaproteobacteria	Desulfovibrionales	Desulfovibrionaceae	Desulfovibrio	intestinalis	0,058	0,067	0,022	1,258	1,123	0,000	0,000
Epsilonproteobacteria	Campylobacterales	Campylobacteraceae	Arcobacter	aquimarinus	0,139	0,413	0,025	1,548	2,366	0,006	0,000
Epsilonproteobacteria	Campylobacterales	Campylobacteraceae	Arcobacter	butzleri	0,795	0,769	0,183	0,952	1,251	0,000	0,000
Epsilonproteobacteria	Campylobacterales	Campylobacteraceae	Arcobacter	cryaerophilus	11,237	10,135	4,013	0,363	0,355	0,017	0,019
Epsilonproteobacteria	Campylobacterales	Campylobacteraceae	Arcobacter	NA	0,564	0,382	0,118	1,964	1,931	0,000	0,000
Epsilonproteobacteria	Campylobacterales	Campylobacteraceae	Arcobacter	NA	0,115	0,285	0,036	1,803	2,728	0,021	0,000
Epsilonproteobacteria	Campylobacterales	Campylobacteraceae	Arcobacter	NA	0,035	0,129	0,008	1,514	2,945	0,068	0,000
Epsilonproteobacteria	Campylobacterales	Campylobacteraceae	Sulfurospirillum	cavolei	0,005	0,090	0,000	2,384	4,425	0,017	0,000
Epsilonproteobacteria	Campylobacterales	Campylobacteraceae	Sulfurospirillum	NA	0,009	0,119	0,003	1,590	3,704	0,010	0,000
Gammaproteobacteria	Aeromonadales	Aeromonadaceae	Aeromonas	NA	0,257	0,007	0,000	5,114	1,647	0,000	0,038
Gammaproteobacteria	Aeromonadales	Aeromonadaceae	Tolumonas	NA	0,183	0,494	0,038	1,744	2,394	0,000	0,000
Gammaproteobacteria	Aeromonadales	Aeromonadaceae	Tolumonas	NA	0,007	0,180	0,002	1,331	4,702	0,093	0,000
Gammaproteobacteria	Enterobacteriales	Enterobacteriaceae	Citrobacter	NA	0,024	0,031	0,004	2,510	3,010	0,015	0,002
Gammaproteobacteria	Enterobacteriales	Enterobacteriaceae	Enterobacter	NA	0,325	0,088	0,040	0,569	0,507	0,048	0,086
Gammaproteobacteria	Enterobacteriales	Enterobacteriaceae	Klebsiella	NA	0,159	0,021	0,010	2,686	1,283	0,000	0,018
Gammaproteobacteria	Pseudomonadales	Moraxellaceae	Acinetobacter	bouvetii	0,052	0,154	0,017	1,641	1,597	0,080	0,091
Gammaproteobacteria	Pseudomonadales	Moraxellaceae	Acinetobacter	haemolyticus	0,024	0,152	0,002	2,857	4,262	0,000	0,000
Gammaproteobacteria	Pseudomonadales	Moraxellaceae	Acinetobacter	johnsonii	0,166	0,184	0,004	4,104	3,683	0,000	0,000
Gammaproteobacteria	Pseudomonadales	Moraxellaceae	Acinetobacter	johnsonii	0,408	0,520	0,147	0,396	0,543	0,065	0,006
Gammaproteobacteria	Pseudomonadales	Moraxellaceae	Acinetobacter	NA	0,046	0,160	0,000	3,475	4,716	0,000	0,000
Gammaproteobacteria	Pseudomonadales	Moraxellaceae	Acinetobacter	NA	0,014	0,428	0,003	1,706	4,335	0,006	0,000
Gammaproteobacteria	Pseudomonadales	Moraxellaceae	Acinetobacter	NA	0,567	0,924	0,187	0,448	0,601	0,026	0,001
Gammaproteobacteria	Pseudomonadales	Pseudomonadaceae	Pseudomonas	NA	0,024	0,066	0,008	1,644	2,285	0,000	0,000
Gammaproteobacteria	Pseudomonadales	Pseudomonadaceae	Pseudomonas	NA	0,128	0,046	0,035	1,440	0,721	0,000	0,031
Gammaproteobacteria	Pseudomonadales	Pseudomonadaceae	Pseudomonas	pseudoalcaligenes	1,274	0,159	0,110	1,143	0,619	0,000	0,005
Gammaproteobacteria	Xanthomonadales	Xanthomonadaceae	Stenotrophomonas	maltophilia	0,018	0,031	0,006	2,004	2,344	0,093	0,042
Bacteria belonging to the phylum Saccharibacteria					0,013	0,021	0,000	3,413	3,467	0,001	0,000
Bacteria belonging to the phylum Saccharibacteria					0,009	0,052	0,000	3,087	4,397	0,000	0,000

Bacteria belonging to the phylum Saccharibacteria					0,028	0,078	0,005	2,116	2,783	0,026	0,002
Bacteria belonging to the phylum Saccharibacteria					0,064	0,068	0,018	1,075	1,172	0,000	0,000
Synergistia	Synergistales	Synergistaceae	Lactivibrio	NA	0,061	0,004	0,001	3,897	1,760	0,000	0,056
Mollicutes	NB1-n	NA	NA	NA	0,005	0,013	0,000	2,051	3,759	0,026	0,000
Verrucomicrobia OPB35_soil_group	NA	NA	NA	NA	0,167	0,013	0,000	5,010	3,761	0,000	0,000

Table S4. Clinical indicator bacteria in influent. 10 of the 207 *clinical enriched taxa* were found to be significant more abundant in influent (I) when compared to community (C) wastewater. 4 of the *clinical indicator bacteria* were found to be more abundant in community wastewater when compared to influent (shown in grey), however, for three species the difference was not significant ($p > 0.05$).

**Bacterial identification**							**Mean abundance (%)**		**Log2-FoldChange**	**p-value**
**Kingdom**	**Phylum**	**Class**	**Order**	**Family**	**Genus**	**Species**	**I**	**C**	**I - C**	
Bacteria	Proteobacteria	Gammaproteobacteria	Aeromonadales	Aeromonadaceae	Tolumonas	NA	0,198	0,038	0,780	0,008
Bacteria	Bacteroidetes	Bacteroidia	Bacteroidales	Prevotellaceae	Prevotella_9	NA	0,153	0,018	0,667	0,040
Bacteria	Proteobacteria	Deltaproteobacteria	Desulfovibrionales	Desulfovibrionaceae	Desulfovibrio	desulfuricans	0,038	0,086	-0,585	0,036
Bacteria	Proteobacteria	Epsilonproteobacteria	Campylobacterales	Campylobacteraceae	Arcobacter	NA	1,940	0,036	1,600	0,000
Bacteria	Bacteroidetes	Bacteroidia	Bacteroidales	Porphyromonadaceae	Paludibacter	NA	0,126	0,002	0,982	0,006
Bacteria	Firmicutes	Negativicutes	Selenomonadales	Veillonellaceae	Selenomonas	lacticifex	0,120	0,006	0,832	0,007
Bacteria	Bacteroidetes	Flavobacteriia	Flavobacteriales	Flavobacteriaceae	Cloacibacterium	normanense	0,272	0,339	-0,438	0,099
Bacteria	Proteobacteria	Gammaproteobacteria	Aeromonadales	Aeromonadaceae	Tolumonas	NA	0,115	0,002	0,980	0,007
Bacteria	Bacteroidetes	Bacteroidia	Bacteroidales	Bacteroidaceae	Bacteroides	stercoris	0,074	0,002	0,835	0,036
Bacteria	Bacteroidetes	Bacteroidia	Bacteroidales	Prevotellaceae	Prevotella_9	NA	0,093	0,011	0,818	0,017
Bacteria	Firmicutes	Bacilli	Lactobacillales	Lactobacillaceae	Lactobacillus	delbrueckii	0,112	0,013	0,940	0,005
Bacteria	Proteobacteria	Epsilonproteobacteria	Campylobacterales	Campylobacteraceae	Arcobacter	aquimarinus	0,759	0,025	1,173	0,000
Bacteria	Proteobacteria	Gammaproteobacteria	Pseudomonadales	Pseudomonadaceae	Pseudomonas	pseudoalcaligenes	0,056	0,110	-0,576	0,059
Bacteria	Proteobacteria	Deltaproteobacteria	Desulfovibrionales	Desulfovibrionaceae	Desulfovibrio	intestinalis	0,010	0,022	-0,751	0,058

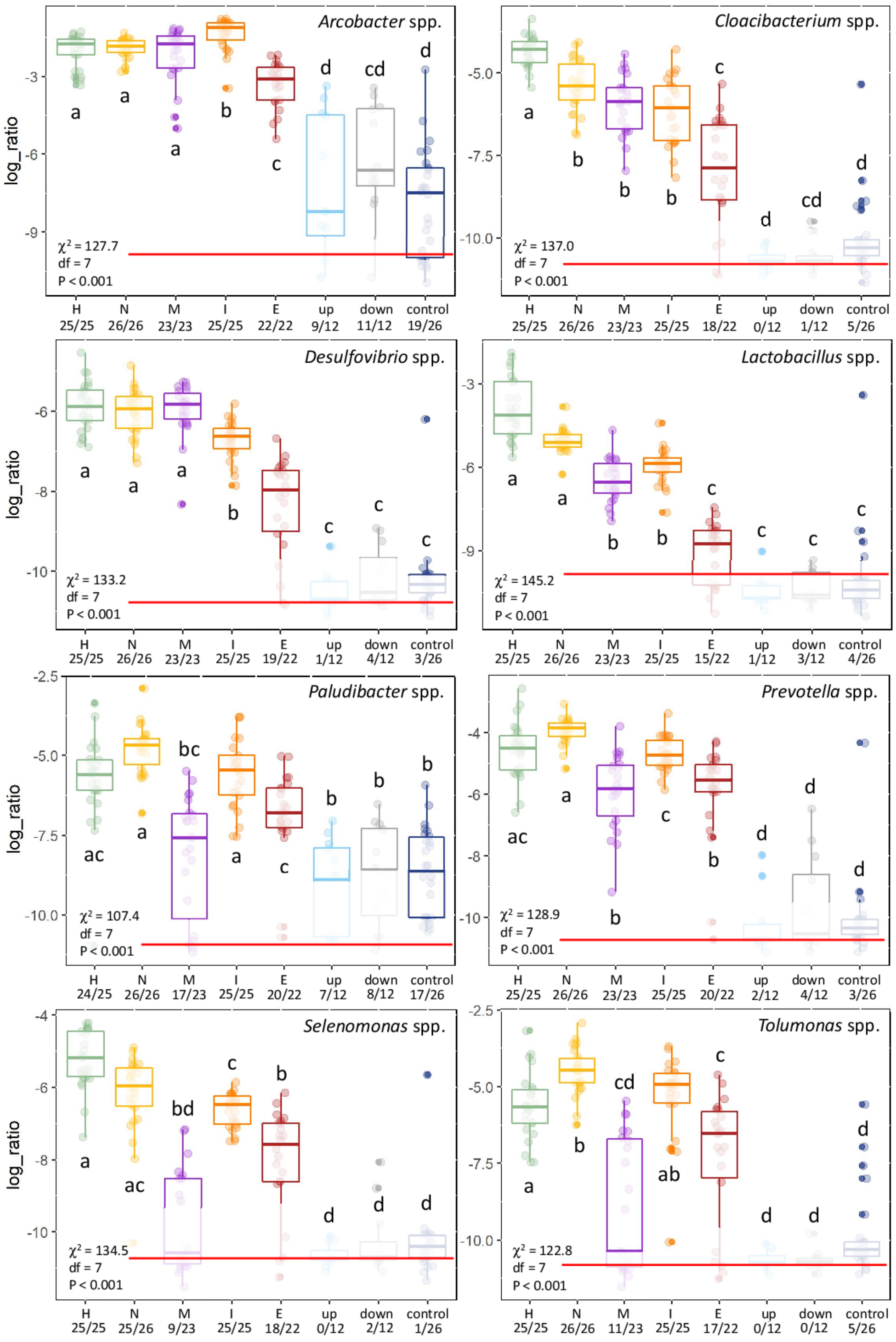

Figure S4. Pathway of genera including clinical enriched genera. Relative abundance of genera along the wastewater pathway. The Kruskal-Wallis statistics are shown on the left side of each panel, for all species there was significant difference in abundance observed between two or more locations. Group differences were assessed Dunn's test with P value adjustment method: BH. The red line represents the average detection limit. Samples below the detection limit are faded. H = hospital, N = nursing home, M = municipal, I = influent, E = effluent, up = upstream surface water, down = downstream surface water, and control = control surface water.

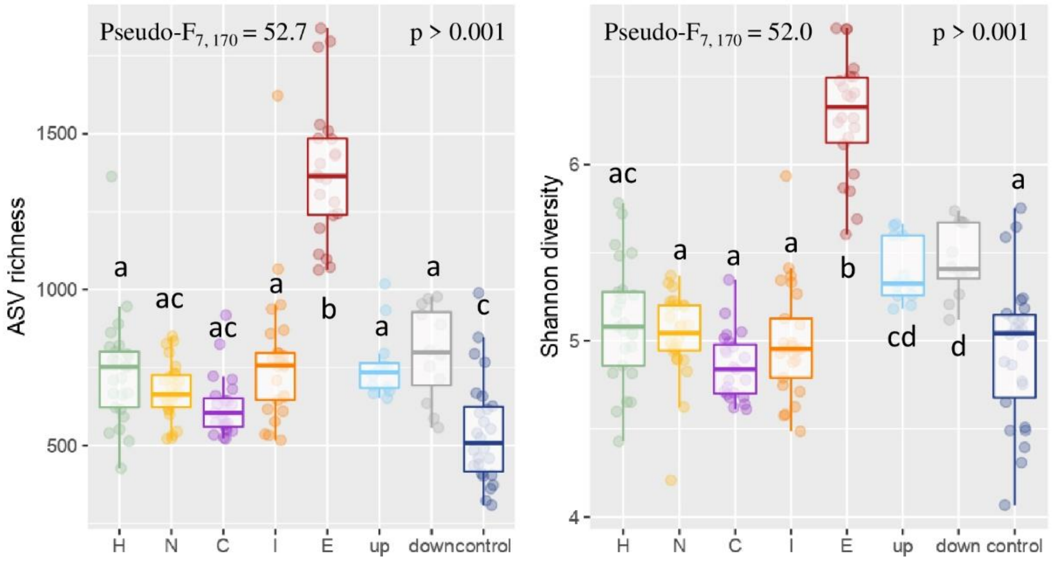

Figure S5. ASV richness and Shannon diversity across all sites. Both ASV richness (left) and Shannon diversity (right) show that effluent was the location with the highest diversity. Group differences were assessed by Tukey HSD. H = hospital, N = nursing home, C = community (municipal), I = influent, E = effluent, Up = upstream surface water, Down = downstream surface water, and Control = control surface water.
